# Drug repurposing in idiopathic pulmonary fibrosis filtered by a bioinformatics-derived composite score

**DOI:** 10.1038/s41598-017-12849-8

**Published:** 2017-10-03

**Authors:** E. Karatzas, M. M. Bourdakou, G. Kolios, G. M. Spyrou

**Affiliations:** 1 Department of Informatics and Telecommunications, University of Athens, 15784 Ilissia Athens, Greece; 2 Center of Systems Biology, Biomedical Research Foundation, Academy of Athens, Soranou Ephessiou 4, 115 27 Athens, Greece; 3 Laboratory of Pharmacology, Department of Medicine, Democritus University of Thrace, Alexandroupolis, Greece; 4 Bioinformatics ERA Chair, The Cyprus Institute of Neurology and Genetics, 6 International Airport Avenue, Nicosia, 2370 Cyprus

## Abstract

Idiopathic Pulmonary Fibrosis (IPF) is a rare disease of the respiratory system in which the lungs stiffen and get scarred, resulting in breathing weakness and eventually leading to death. Drug repurposing is a process that provides evidence for existing drugs that may also be effective in different diseases. In this study, we present a computational pipeline having as input a number of gene expression datasets from early and advanced stages of IPF and as output lists of repurposed drugs ranked with a novel composite score. We have devised and used a scoring formula in order to rank the repurposed drugs, consolidating the standard repurposing score with structural, functional and side effects’ scores for each drug per stage of IPF. The whole pipeline involves the selection of proper gene expression datasets, data preprocessing and statistical analysis, selection of the most important genes related to the disease, analysis of biological pathways, investigation of related molecular mechanisms, identification of fibrosis-related microRNAs, drug repurposing, structural and literature-based analysis of the repurposed drugs.

## Introduction

IPF is a rare, incurable disease of the respiratory system during which fibrotic tissue and scars appear in the lungs. It leads to death within 2–5 years after the diagnosis. Early diagnosis is poor due to the nonspecific symptoms of the disease. Clinical symptoms consist of dyspnea on exertion, dry cough and velcro-like auscultatory. A high resolution computed tomography (HRCT) of the patient’s lungs is needed to differentiate IPF from other idiopathic interstitial pneumonias. Finally, a biopsy of the fibrotic areas from the inflammatory parts of the lung epithelium is needed to accurately determine the existence of IPF. There are different stages of IPF usually labeled as mild or severe. In our study, we refer to mild cases as early, stable or slow and to severe cases as advanced, acute or rapid in accordance to each dataset’s samples. New methods of IPF staging have been recently developed based on gender, age and lung physiology where, given the required measurements, the probability of mortality for the patient in the next 3 years is calculated^1^. Molecular mechanisms of IPF have been studied before, including cellular interactions via a complex cytokine-signalling mechanism, heightened collagen gene expression, signaling events that mediate fibroblast proliferation and myofibroblasts, cell matrix interactions^2^, endoplasmic reticulum stress, shortened telomeres, inflammation and immune mechanisms, oxidative stress and signaling and procoagulant mechanisms^3^. There are currently two FDA approved drugs with inhibiting role against IPF; nintedanib and pirfenidone. Despite that, an actual treatment that completely cures the patient from the disease remains to be found.

Other studies suggest that inhaled interferon gamma aerosol may pose as an effective treatment against IPF. An 80-week treatment of inhaled interferon-gamma for 10 patients showed significant decrease in profibrotic cytokines and reversed the decrease in lung capacity and diffusing capacity for carbon monoxide^4^. Recent research proposes that the guidelines for diagnosis, prognosis and treatment of IPF should be targeting individuals in a personalized medicine approach while making use of multi-omics (genomics, proteomics, metabolomics, microbiomics, etc.) training data sources^5^.

Drug repurposing is the process during which known drugs are applied to different diseases. Using drug repurposing, we avoid the high cost of developing entirely new drugs. In silico drug repurposing in particular, further speeds up the process and reduces the cost, as it results in computationally ranked lists of repurposed drugs for a disease. The use of drug repurposing in rare or orphan diseases such as IPF is very important as it may lead to important connections between the disease and existing drugs^6^. Drug repurposing studies have been previously published on other diseases like Alzheimer, where Siavelis *et al*.^7^ used a bioinformatics pipeline to discover potential inhibitor drugs against Alzheimer’s disease. More drug repurposing studies have been published on malaria and tuberculosis^8^, on other parasitic diseases such as trypanosomiasis, toxoplasmosis, cryptosporidiosis and leishmaniasis^9^, on small cell lung cancer^10^ and on gastrointestinal stromal tumor^11^ as well as on breast cancer^12^. To the best of our knowledge, *in silico* drug repurposing studies targeting IPF haven’t been mentioned before. Nevertheless, recent biological pathway-related drug repurposing studies for IPF suggest promising results. These studies perform experiments *in vitro* on human cell lines with IPF (PI3K inhibition)^13,14^ as well as *in vivo* on mouse models with IPF (LTB4 inhibition)^15^.

This study’s main contribution is the presentation of a bioinformatics pipeline for computational drug repurposing that ends with re-ranking of the repurposed drugs according to a composite drug repurposing score (CoDReS). This score aims to combine the classical drug repurposing inhibition score with other major components related to the suitability of a drug/chemical compound to be successfully applied to the disease under study. These extra components are the structural druglikeness, the functional implication to the disease and the severity of side effects for each drug. Moreover, the present study is targeting IPF through the aforementioned computational drug repurposing pipeline and concludes to candidate drugs (some of which are also natural products), significant genes, microRNAs and pathways functionally related to IPF. Specifically, we start from analyzing gene expression datasets on various stages of IPF concluding to properly ranked candidate drugs that might inhibit IPF via drug repurposing. Moreover, we use the top differentially expressed genes, the resulted repurposed drugs along with their targets and fibrosis-related microRNAs to better understand the mechanisms and the biological pathways perturbed by IPF.

## Methods

### Datasets

We searched NCBI (http://www.ncbi.nlm.nih.gov/geo/) for gene expression datasets containing healthy and IPF samples, without time periods or dosage, using the query:

“idiopathic pulmonary fibrosis”[Title] AND “Homo Sapiens”[Organism] AND “Expression profiling by array”[Filter] AND “Normal”[All Fields] AND “tissue”[All Fields] NOT “therapy”[All Fields] NOT “treatment”[All Fields] NOT “Drug”[All Fields].

We then kept all data coming from lung tissue and removed data either coming from blood analysis or containing replications. Finally, we filtered the results to correspond to platforms of Affymetrix, Illumina and Agilent containing at least 10 RNA samples. Until October 27th 2015, the query results induced 3 gene expression datasets: GSE10667^16–18^, GSE24206^19^ and GSE44723^20^ (Table 1). Specifically, GSE10667 contains 15 control samples derived from normal lung tissues obtained from disease-free margins with normal histology of lung cancer resection, 23 samples from lung tissue with usual interstitial pneumonia and 8 samples from lung tissue with acute exacerbations. Samples from GSE24206 include 6 normal samples derived from lung tissues of healthy donors, 8 biopsy samples from patients with early IPF and 9 explant samples from patients of an advanced stage of IPF. Finally, samples from GSE44723 consist of 4 normal samples from cultured human fibroblasts without fibrosis, 6 samples from cultured human fibroblasts with slow progressing fibrosis and 4 samples from cultured human fibroblasts with rapid progressing fibrosis.

**Table 1:** Details of the gene expression datasets.

GEO accesion number	Total Samples	IPF stage 1	IPF stage 2	Normal
GSE44723	14	6	4	4
GSE24206	23	8	9	6
GSE10667	46	23	8	15

### Gene selection

We performed statistical analysis on the aforementioned gene expression datasets using R/Bioconductor package Limma^21–23^. We selected the top 150 up-regulated and top 150 down-regulated genes (p-value < 0.05), due to the input restrictions of the drug repurposing tool - Cmap, of each dataset regarding their fold change (FC). To introduce a second, independent way to prioritize the most important genes related to IPF per dataset, we used Netwalker^24^, as a random walk-based approach, to construct a second list with the same number of up and down regulated genes.

### Drug repurposing and scoring formula

We separately used the two lists of up and down regulated genes of each dataset as input in three drug repurposing tools (during December 2015): connectivity map (Cmap)^25^ (https://www.broadinstitute.org/cmap/), LINCS (http://apps.lincscloud.org/query) and Spiedw^26^ (http://www.spied.org.uk/cgi-bin/wSPIED.cgi). Cmap contains a database of genome-wide transcriptional expression data coming from human cell lines that have been treated by drugs. Each drug in combination with each cell line gives a specific gene signature. Given a list of probes and using matching algorithms, cmap returns scored drug lists that propose potential inhibitors or activators of the perturbation. At the time of use (December 2015), Cmap was consisting of more than 7000 gene signatures, 1309 drugs and 5 cancer cell lines. LINCS, the expansion of the Cmap project, was consisting of 476251 signatures, 20143 molecules and 13 cell lines. Spiedw was gathering about 200000 gene signatures from experiments on human, mouse and rat samples, across the platforms of Affymetrix, Illumina and Agilent. We kept the drugs with inhibiting score ≤−0.5 found in at least 2 out of the 3 tools and then we created a union list of drugs for each dataset. Finally, we kept all drugs that were found in at least 2 out of the 3 datasets of the same stage of the disease.

All repurposed drugs from this point on, have been considered as potential candidates for the treatment of IPF. We created a list of all repurposed drugs, disregarding the stage in which each drug was discovered, and devised a novel scoring formula in order to rank all the potential IPF inhibitors. In order to feed properly the composite scoring formula, we calculated and normalized the scores concerning inhibition, structural and functional properties as well as potential side effects.

Inhibition scores result from each of the three drug repurposing tools per experiment. We calculated the corresponding score to each drug by aggregating the total inhibition scores from every drug repurposing tool (as long as the score is below −0.5), for both the Limma and Netwalker analysis for each stage of IPF. Then we normalized the results by dividing with the max drug-score for each stage.

In the sequel, we performed calculations regarding the structural druglikeness, the functional implication to the disease and the severity of side effects for each drug.

Structural druglikeness is measured via SwissADME^27^ (http://www.swissadme.ch/), an online tool which takes as input the structural description (simplified molecular-input line-entry system: SMILES) of a drug and returns (among other chemical properties) the number of rules that might be violated by each chemical substance in order for it to be used as a drug. For each rule violated by a drug, we increment its penalty score and then we normalize the scores by dividing with the number of maximum rules violated by a single drug. Then, we take this value’s distance from 1, since we need to give the highest score of 1 to the drugs with no violations at all. To measure the functional score of each drug, we used the online tool ToppGene^28^ (https://toppgene.cchmc.org/prioritization.jsp). We inserted a list of 71 IPF unique gene identifiers, as found in Malacards^29^ (http://www.malacards.org/), as a train set and then checked the gene targets of each repurposed drug as test set against it, selecting Pathway, Interaction and Coexpression enrichment between the two lists. Each experiment returns a reranked gene-target list with a normalized score. We selected the average of each score list to act as the functional score for each repurposed drug. To normalize the results, we divided all values by the maximum value observed.

Finally, through Drugs.com’s Side Effects webpage (https://www.drugs.com/sfx/), we devised a score for each drug regarding its potential side effects. There are different categories for the various side effects ranging from major and common side effects to minor side effects with unknown incident. We created a formula to give each drug a specific score according to its various side effects. There are eight total classes of importance. For each of them we give an incremental weight ranging from one to eight respectively.

The scoring formula regarding side effects is considered as:

$$\sum _{i=1}^{8}Nclass(i)\times i$$

where Nclass(i) is the number of side effects for each drug in the class i.

Then we are dividing by the max value to normalize all drug side effect scores and subsequently we are taking this value’s distance from 1, since we need to give the higher scores to drugs with less side effects.

Compiling all the aforementioned partial scores, we propose the following calculation for a composite drug repurposing score (CoDReS):

$${\rm{CoDReS}}={{\rm{W}}}_{{\rm{is}}}\,\times \,{\rm{InhibScore}}+{{\rm{W}}}_{{\rm{ss}}}\,\times \,{\rm{StructScore}}+{{\rm{W}}}_{{\rm{fs}}}\,\times \,{\rm{FuncScore}}+{{\rm{W}}}_{{\rm{ses}}}\,\times \,{\rm{SideEffectsScore}}$$

The weights of the components comprising the CoDReS score can be derived either by following optimization approaches focused on ground-truth data or by following drug-mining strategies focused on qualitative criteria. In the current study, we followed the latter approach; we decided to downgrade the initial drug repurposing inhibition score that presents an initial potentiality of the drug to be a good candidate, and upgrade the contributions from structural, functional and pharmacological insights related to the suitability of the drug to be a success story. Therefore, the weights we used for each experiment were the following:

$${{\rm{W}}}_{{\rm{is}}}=0{.4,{\rm{W}}}_{{\rm{ss}}}=0{.2,{\rm{W}}}_{{\rm{fs}}}=0{.2,{\rm{W}}}_{{\rm{ses}}}=0.2$$

This balance between drug potentiality (currently W_is_ = 0.4) and drug suitability (currently W_ss_ + W_fs_ + W_ses_ = 0.6) reflects a specific drug ranking strategy. In the absence of sufficient number of training/test datasets containing drugs and drug efficiencies against the selected disease, a usual case in drug discovery/repurposing investigations, it is on the researcher’s decision to select the proper drug ranking strategy.

Finally, we present structural clusters of the repurposed drugs for each stage-experiment, based on the hierarchical clustering algorithm of ChemBioServer^30^ (http://bioserver-3.bioacademy.gr/Bioserver/ChemBioServer/). The top ranked repurposed drugs are presented for each cluster and stage of the disease.

### Pathway analysis and targeted genes

We used the gene lists (up and down regulated) of each experiment as input to Enrichr’s (http://amp.pharm.mssm.edu/Enrichr/)^31^ KEGG^32–34^ pathway analysis in order to discover and point out molecular mechanisms involved in IPF. We unified the resulted pathway lists of each dataset’s results (from both Limma and NetWalker) and then calculated the pathways intersection between the 3 datasets of the same stage analysis of the disease. We then found the genes contained in each pathway as well as the genes targeted by the resulted repurposed drugs. Drug gene targets were found in SuperTarget^35^ and DrugBank^36–38^. SuperTarget contains a database of information on about 332828 drug-target interactions and DrugBank is another database containing bioinformatics and cheminformatics details on 8174 drugs and their targets.

### Structural analysis and natural compounds

We downloaded the structure files (in mol or sdf format) of the resulted drugs from DrugBank, Chemspider^39^ (http://www.chemspider.com/) and PubChem^40,41^ (https://pubchem.ncbi.nlm.nih.gov/).We then used the OpenBabel^42^ software to convert all these files to a single sdf library file for each experiment which was then used as an input in the ChemBioServer. Through ChemBioServer we performed clustering based on the chemical and structural similarity of the drugs. We finally pointed out which of the resulted drugs are either natural compounds or structurally similar to natural compounds found in SuperNatural II and bibliography.

**Figure 1:**
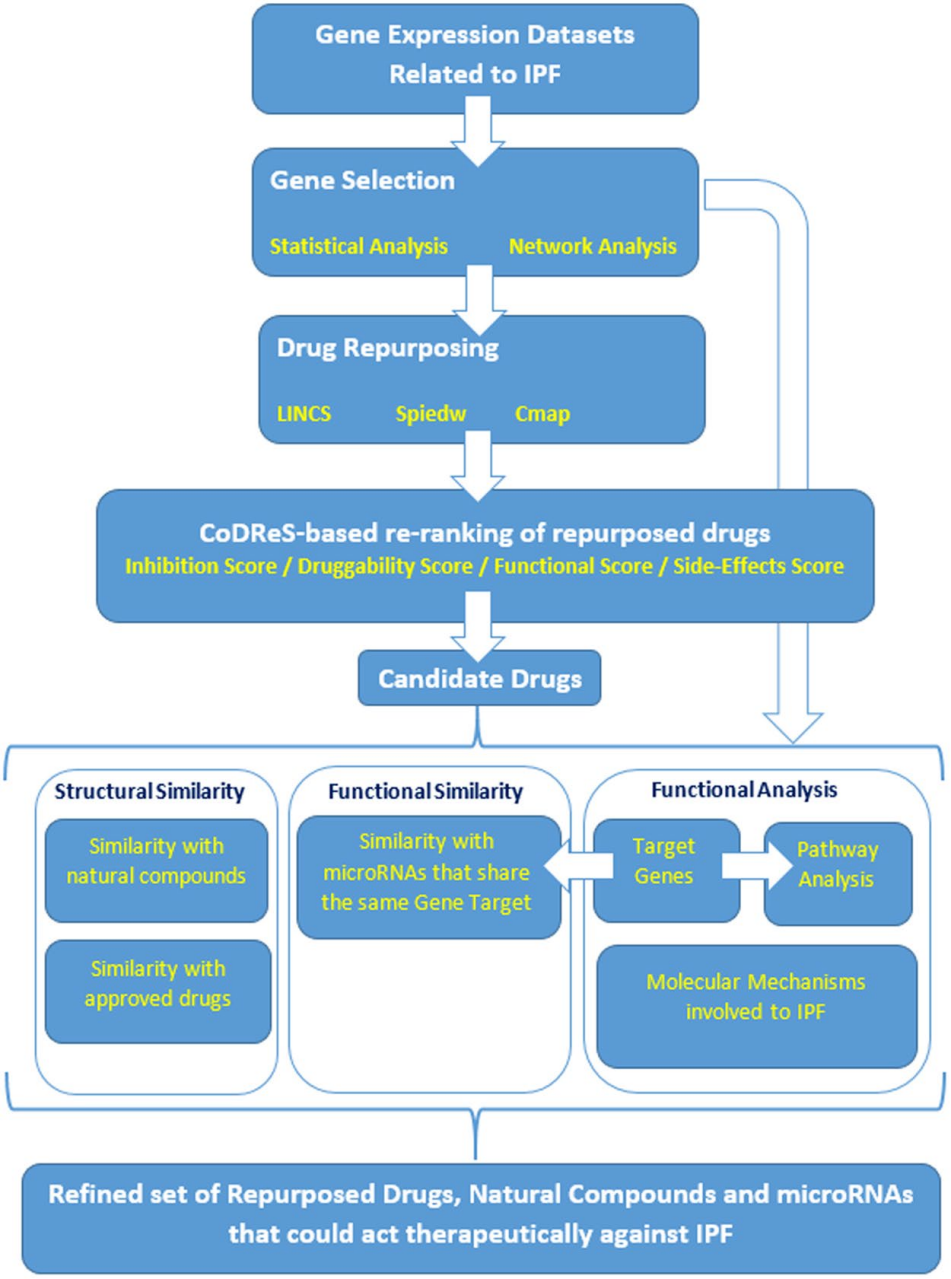
Layout of the complete pipeline.

A layout of the complete pipeline is shown in Fig. 1.

### Data availability

All links to the data analysed during this study are included in this published article.

All data generated during this study are included in this published article (and its Supplementary Information files).

The intermediate datasets of the pipeline generated during the current study are available from the corresponding author on reasonable request.

### Image acquisition and processing

The first figure was created with Microsoft Word. Figures containing Venn diagrams were acquired from the web tool Venny (http://bioinfogp.cnb.csic.es/tools/venny/). Figures displaying structural clustering were acquired by ChemBioServer and figures with networks were created using the Cytoscape software (http://www.cytoscape.org/). For the processing of images to correspond to 300dpi we used the software IrfanView (http://www.irfanview.com/).

## Results

### Gene lists

Using gene selection methods as described in the Materials and Methods section, we resulted into two gene lists per dataset, derived from Limma’s statistical analysis and from NetWalker’s random walk approach. All gene lists are shown in Supplementary Table 1. To conclude in the most important genes related to each stage of the disease, we combined the two gene lists per dataset, creating a unified up-regulated list and a unified down-regulated list per dataset. Finally, we integrated the information from all datasets by building up- and down-regulated gene lists separately, including genes that are common in at least two datasets for each stage of the disease. All lists containing the important up- and down-regulated genes for each stage are shown in Supplementary Table 2. Genes that were found in all three datasets of a stage are highlighted in light gray.

### Repurposed Drugs

Using the tools and methods for drug repurposing as described in the materials and methods section, we concluded in 5 drugs for the IPF vs normal series of experiments, 7 drugs for stage 2 IPF vs normal samples, 10 drugs for stage 1 vs normal and 6 drugs for stage 2 vs stage 1 IPF. Drugs are shown in Table 2.

**Table 2:** Drug results for each stage of the disease.

IPF vs Normal	Stage 2 vs Normal	Stage 1 vs Normal	Stage 2 vs Stage 1
thapsigargin	thapsigargin	thapsigargin	norethisterone
lycorine	puromycin	anisomycin	alsterpaullone
carbimazole	anisomycin	perphenazine	hesperetin
anisomycin	lasalocid	lycorine	azacitidine
perphenazine	etoposide	naltrexone	clobetasol
	irinotecan	spiradoline	acacetin
	niclosamide	colistin	
		benzocaine	
		rotenone	

The conventional use of each drug is presented in Table 3.

**Table 3:** Conventional use of repurposed drugs.

Repurposed drugs	Conventional Use
thapsigargin	Tumor promoter in mammalian cells and useful in experimentation examining the impacts of increasing cytosolic calcium concentrations. Also used in traditional medicine as a counter-irritant
lycorine	Inhibits protein synthesis and has promising activities such as antibacterial, antiviral or anti-inflammatory effects. Also exhibits antimalarial activity
carbimazole	Used to treat hyperthyroidism
anisomycin	Inhibits protein and DNA synthesis. Also mentioned as potential psychiatric drug and has been proposed for selective removal of memories by injection into the hippocampus
perphenazine	Antipsychotic drug with sedating and anxiolytic properties
puromycin	Used in cell biology as a selective agent in cell culture systems
lasalocid	Used as a coccidiostat, especially in poultry
etoposide	Chemotherapy medication for the treatment of various types of cancer
irinotecan	Used to treat colon cancer and small cell lung cancer
niclosamide	Used to treat tapeworm infestations
naltrexone	Used to treat opioid addiction, alcoholism and obesity
spiradoline	Used to cause sedation, along with analgesic and diuretic effects but stopped being clinically used due to side effects such as dysphoria and hallucinations
colistin	Used to treat infections caused by Pseudomonas, Escherichia, and Klebsiella species but has high kidney toxicity
benzocaine	Used as a topical pain reliever or in cough drops. Combined with antipyrine to relieve ear pain and remove ear wax
rotenone	Used as a broad-spectrum insecticide, piscicide, and pesticide
norethisterone	Used in hormonal contraceptives, hormone replacement therapy and in the treatment of gynecological disorders
alsterpaullone	Has been used to modify neuropathological events associated with Alzheimer’s disease and to alter cell proliferation or protein expression in various diseases
hesperetin	Appears to reduce cholesteryl ester mass and may have antioxidant, anti-inflammatory, anti-allergic, hypolipidemic, vasoprotective and anticarcinogenic actions
azacytidine	Used in the treatment of myelodysplastic syndrome
clobetasol	In the form of lotion and shampoo, it is used to treat skin conditions that have not responded well to other corticosteroids, including severe psoriasis and eczematous dermatitis.
acacetin	Has anti-peroxidative and anti-inflammatory properties and used in traditional Chinese medicine. Recognized as an antiproliferative agent on cancer cell lines

**Figure 2:**
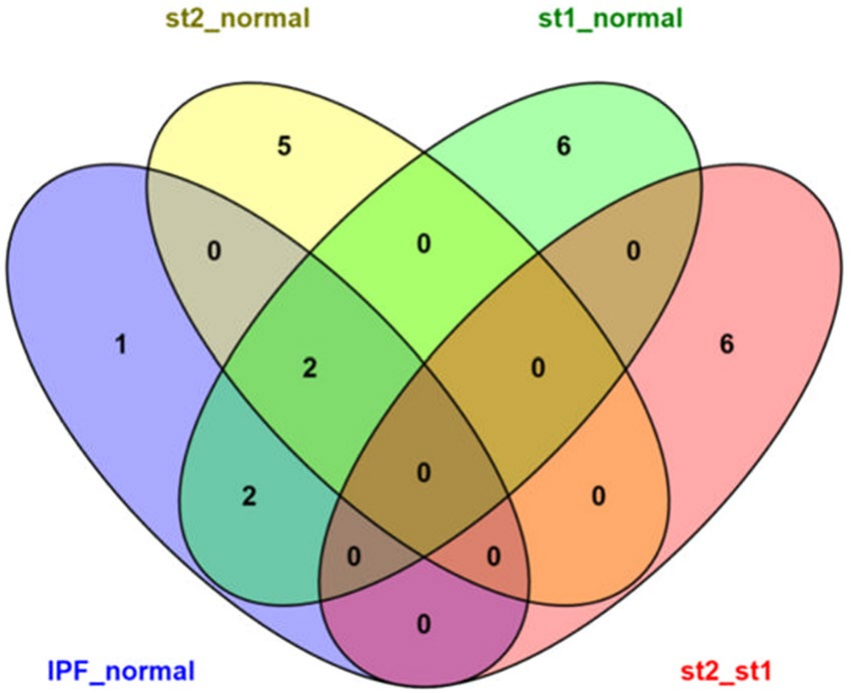
Venn diagram between final drugs of each stage of IPF.

Some of the drugs exist in more than one phases of the disease. A Venn diagram was constructed in order to point out the common drugs between the different stages of the disease (Fig. 2). Thapsigargin and anisomycin seem to be useful during the transition of normal lungs to fibrotic lungs during both stages 1 and 2 of the disease. Thapsigargin though, is known to induce endoplasmic reticulum (ER) stress, which worsens the fibrosis^43,44^. On the other hand, anisomycin inhibits the induction of ER stress^45^, hence might be a good competitor against IPF. Lycorine and perphenazine are contained in two out of the four experiments; from normal lungs to fibrotic in general as well as from normal lungs to stage 1 fibrosis. No similarities between the drugs of the stage 1 to stage 2 transition of the disease and the rest of the experiments were found. There are also some past references to puromycin and acacetin in relation to IPF. Puromycin is known to target inflammatory cells even though they might not be a disorder of IPF as suggested by Ying Dong Xu *et al*.^46^ while acacetinalleviates telomere position effect (TPE) in human cells. There is mounting evidence that telomeres are involved in idiopathic pulmonary fibrosis^47^ especially on the division phase of the cell cycle^41^. Niclosamide has been mentioned to reverse fibrosis on the skin and lungs of mice that had Systemic Sclerosis with pulmonary fibrosis^48^. Niclosamide has a well-documented safety profile and its use can be considered for clinical trials.

After finding all repurposed drugs, we calculated their structural similarity with the FDA approved drugs for the disease, namely nintedanib and pirfenidone, using ChemBioServer (Supplementary Figures 1–4). We observe that there is diversity in the structural similarity of the repurposed drugs to nintedanib and pirfenidone. This can be interpreted both ways: the repurposed drugs that are structurally similar to nintedanib or pirfenidone (irinotecan, puromycin) may have a similar mode of action but may be advantageous compared with the existing ones, whereas the repurposed drugs that are not similar with nintedanib or pirfenidone (benzocaine, norethisterone) may act on other pathways exploiting different mechanisms.

Interestingly, some of the repurposed drugs are found as natural compounds (Table 4).

**Table 4:** Repurposed Drugs that are natural compounds.

Drug	Chemical Compound	Common source	Genus	Family	Kingdom
thapsigargin	sesquiterpene lactone	Thapsia garganica	Thapsia	Apiaceae	Plantae
lycorine	toxic crystalline alkaloid	bush lily (Clivia miniata), surprise lilies (Lycoris), daffodils (Narcissus)	Clivia, Lycoris, Narcissus	Amaryllidaceae	Plantae
anisomycin	antibiotic	Streptomyces griseolus	Streptomyces	Streptomycetaceae	Bacteria
puromycin	aminonucleoside antibiotic	Streptomyces alboniger	Streptomyces	Streptomycetaceae	Bacteria
colistin	polymyxin antibiotic	Paenibacillus polymyxa	Paenibacillus	Paenibacillaceae	Bacteria
rotenone	crystalline ketonic	seeds and stems of Pachyrhizus erosus, roots of several members of Fabaceae	Pachyrhizus	Fabaceae	Plantae
hesperetin	bioflavonoid (flavanone)	citrus flavanoids: hesperidin, quercitrin, rutin and the flavone tangeritin	Citrus	Rutaceae	Plantae
acacetin	O-methylated flavone	black locust (Robinia pseudoacacia), damiana (Turnera diffusa), silver birch (Betula pendula) and the fern Asplenium normale	Robinia, Turnera, Betula, Asplenium	Fabaceae	Plantae

Irinotecan is a semisynthetic analogue of the natural alkaloid camptothecin, which is isolated from the bark and stem of Camptothecaacuminata from family Cornaceae (Nyssaceae) commonly known as happy tree, cancer tree or tree of life. Niclosamide is a synthetic teniacide. Natural taeniacides include pumpkin seed and cucumber from the family of Cucurbitaceae and pomegranate from the family of Lythraceae. Finally, Clobetasol propionate is a corticosteroid of the glucocorticoid class used to treat various skin disorders including eczema and psoriasis. It’s not a natural compound but can be found in shampoo, mouse, ointment and emollient cream presentations.

### Scoring Formula

In this section of the study we present our new scoring formula in order to rank the potential repurposed drugs for each stage of the disease. Structural, functional and side effects’ scoring deal with general aspects of the drugs and are not stage-specific. Based on the scoring methodology of Methods section, the general structural, functional and side effects’ scores are presented in Table 5. We note that for the substances with unknown side effects we set their side effects’ score to zero.

**Table 5:** Structural, Functional and Side Effects’ Scores.

Repurposed Drugs	Structural: Druglikeness	Functional: ToppGene enrichment (interactions, pathways, coexpressions)	Side effects
thapsigargin	0.250	0.384	0.000
lycorine	1.000	0.668	0.000
carbimazole	0.938	0.554	0.000
anisomycin	1.000	0.459	0.000
perphenazine	1.000	0.569	0.000
puromycin	0.750	0.459	0.000
lasalocid	0.500	0.000	0.000
etoposide	0.438	0.518	0.504
irinotecan	0.750	0.572	0.000
niclosamide	0.938	0.361	0.973
naltrexone	1.000	0.699	0.829
spiradoline	1.000	0.769	0.000
colistin	0.000	0.000	0.000
benzocaine	0.938	0.427	0.000
rotenone	1.000	0.263	0.000
norethisterone	1.000	0.523	0.000
alsterpaullone	1.000	1.000	0.000
hesperetin	1.000	0.411	0.000
azacitidine	0.750	0.609	0.128
clobetasol	1.000	0.692	0.000
acacetin	1.000	0.312	0.000

Table 6 shows the normalized scores of each repurposed drug regarding the Limma and NetWalker experiments of each stage of the disease, for datasets GSE10667, GSE24206 and GSE44723 as well as their final CoDReS score and their re-ranking position shift.

**Table 6:** Normalized Inhibition score, CoDReS score and re-ranking position shift of the repurposed drugs per stage.

IPF vs Normal				Stage 1 vs Normal			
Repurposed drugs	Normalized inhibition score	Sorted CoDReS	Re-ranking shift	Repurposed drugs	Normalized inhibition score	Sorted CoDReS	Re-ranking shift
lycorine	0.915	0.7	+2	naltrexone	0.55	0.726	+7
perphenazine	0.949	0.693	0	anisomycin	1	0.692	−1
carbimazole	0.846	0.637	+1	lycorine	0.656	0.596	+3
anisomycin	0.793	0.609	+1	carbimazole	0.736	0.593	0
niclosamide	0.331	0.587	+6	perphenazine	0.622	0.563	+2
puromycin	0.741	0.538	0	niclosamide	0.266	0.561	+7
thapsigargin	1	0.527	−6	benzocaine	0.693	0.55	−2
alsterpaullone	0.16	0.464	+5	spiradoline	0.385	0.508	+4
rotenone	0.527	0.463	0	thapsigargin	0.853	0.468	−6
spiradoline	0.231	0.446	+2	puromycin	0.539	0.457	0
benzocaine	0.394	0.431	−1	rotenone	0.484	0.446	0
lasalocid	0.706	0.382	−4	colistin	0.935	0.374	−10
azacitidine	0.108	0.341	+1	azacitidine	0.086	0.332	+1
etoposide	0.108	0.335	+1	lasalocid	0.542	0.317	−5
colistin	0.726	0.29	−8	irinotecan	0.083	0.298	0
**Stage 2 vs Normal**				**Stage 2 vs Stage 1**			
anisomycin	0.92	0.66	+2	niclosamide	0.769	0.762	+4
niclosamide	0.502	0.655	+4	alsterpaullone	0.809	0.724	+2
etoposide	0.7	0.572	+2	azacitidine	1	0.697	−2
alsterpaullone	0.346	0.538	+9	irinotecan	0.972	0.653	−2
puromycin	0.718	0.529	−1	clobetasol	0.743	0.636	+2
thapsigargin	1	0.527	−5	etoposide	0.855	0.634	−3
perphenazine	0.489	0.509	0	naltrexone	0.281	0.618	+7
lycorine	0.372	0.482	+2	perphenazine	0.73	0.606	0
lasalocid	0.937	0.475	−7	norethisterone	0.744	0.602	−3
irinotecan	0.466	0.451	−2	puromycin	0.689	0.517	−1
carbimazole	0.365	0.444	0	hesperetin	0.539	0.498	0
rotenone	0.436	0.427	−3	acacetin	0.567	0.489	−2
spiradoline	0.117	0.401	+3	anisomycin	0.427	0.462	−1
azacitidine	0.187	0.372	+1	spiradoline	0.092	0.391	+5
benzocaine	0.198	0.352	−1	carbimazole	0.111	0.343	+3
hesperetin	0.115	0.328	+1	rotenone	0.18	0.324	0
colistin	0.356	0.142	−5	benzocaine	0.123	0.322	0
				thapsigargin	0.318	0.254	−5
				lasalocid	0.215	0.186	−4

**Figure 3:**
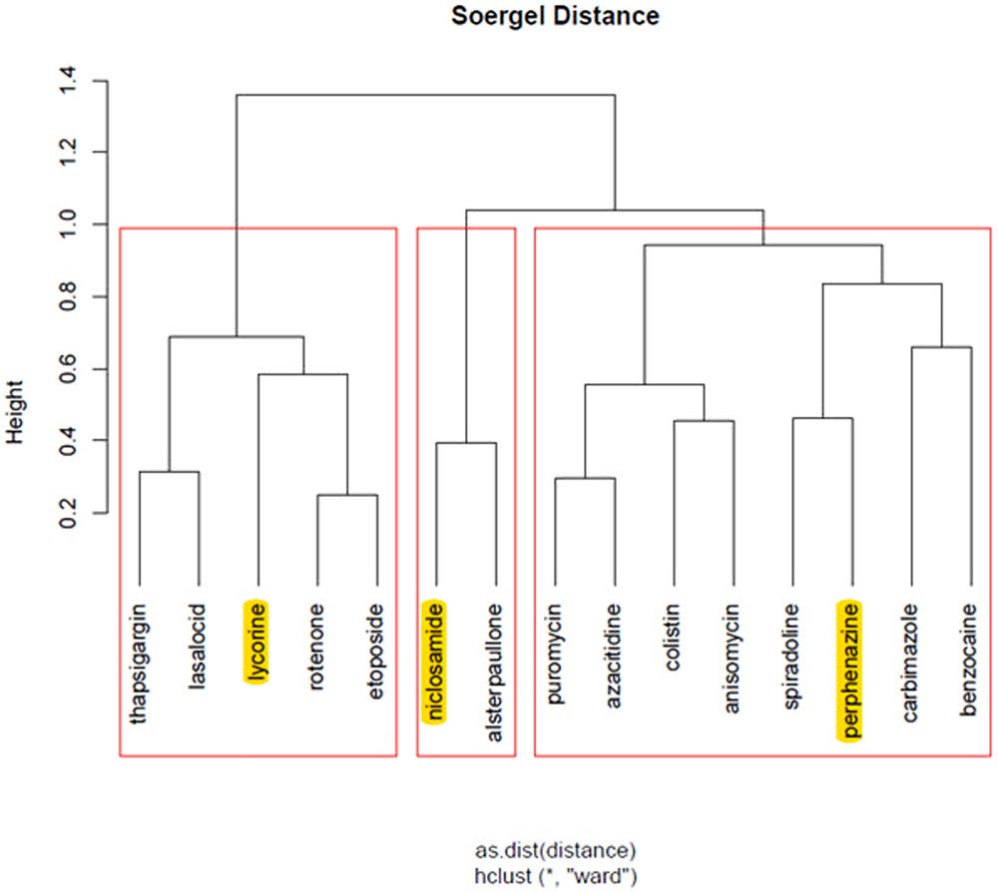
Hierarchical clustering and top scored repurposed drugs-IPF vs Normal.

**Figure 4:**
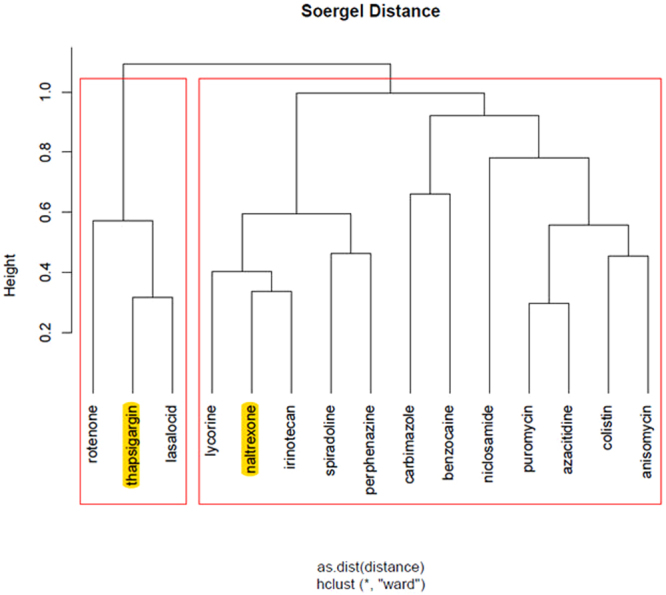
Hierarchical clustering and top scored repurposed drugs-stage 1 vs Normal.

**Figure 5:**
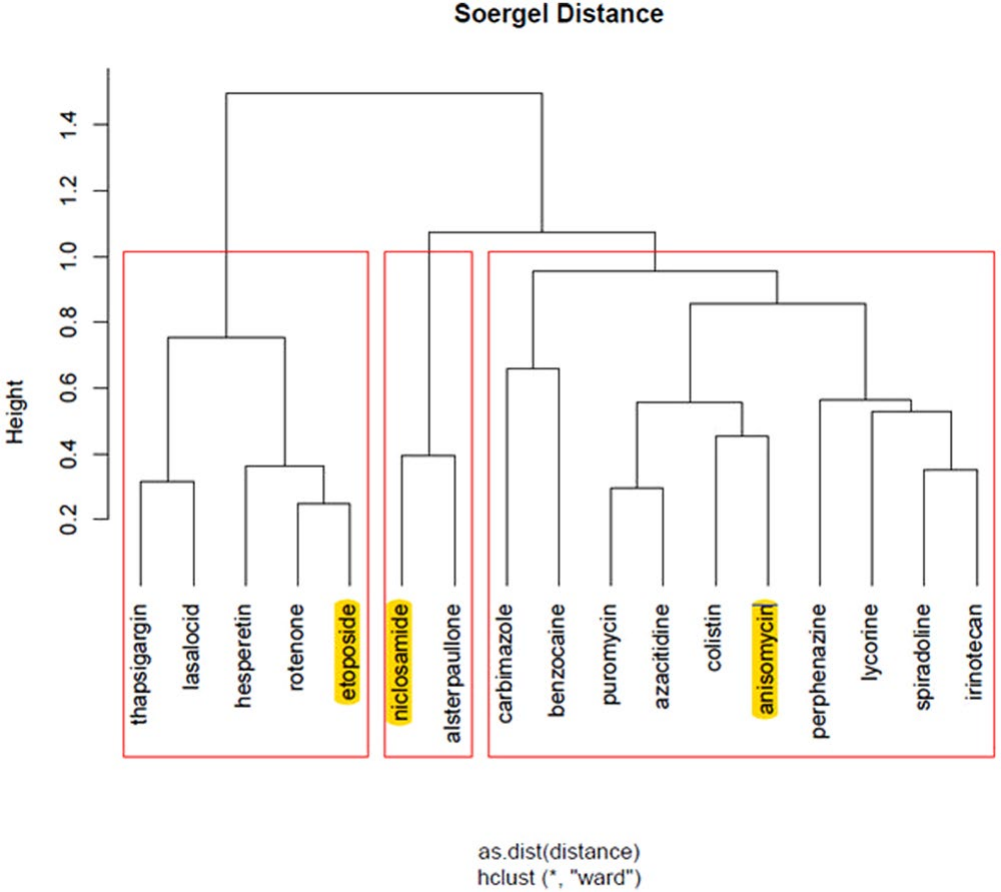
Hierarchical clustering and top scored repurposed drugs-stage 2 vs Normal.

**Figure 6:**
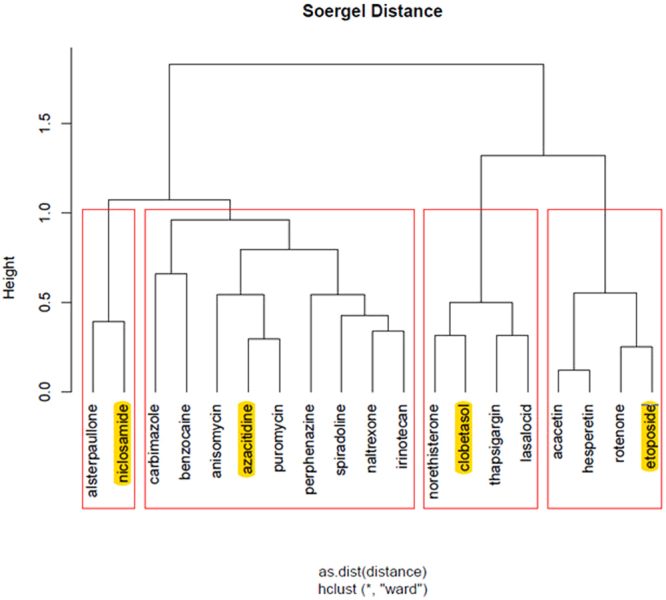
Hierarchical clustering and top scored repurposed drugs-stage 2 vs stage 1.

Finally, we highlight the top ranked drugs of the hierarchical clusters, for each stage of the disease, as returned from ChemBioServer in Figs 3–6. This way, we can conclude to the CoDReS top ranked drugs that belong to different structural clusters and thus they represent different structural families and possibly different modes of action. Thus, we believe that the drugs shown in Table 7 should have priority in experimentation with bioassays and/or animal models.

**Table 7:** Drugs that are both highly scored and structural representatives for each stage.

IPF vs Normal	Stage 2 vs Normal	Stage 1 vs Normal	Stage 2 vs Stage 1
lycorine	etoposide	thapsigargin	niclosamide
niclosamide	niclosamide	naltrexone	azacitidine
perphenazine	anisomycin		clobetasol
			etoposide

### Validation of CoDReS scoring schema

The CoDReS scoring scheme is different from the usual drug repurposing inhibition score in the aspect that it provides extended information about the repurposed drugs, regarding crucial aspects such as how feasible will be the substances to act as drugs, how dangerous their side effects might be and how similar each of the drugs’ target gene lists are to gene lists related to the disease under study, in our case IPF. Other studies have tried to give additional information in drug repurposing results, such as making use of DrugBank’s therapeutic categories by measuring structural and functional distances between drugs of different categories^49^. CoDReS also provides structural and functional information, next to the inhibition score, as well as it incorporates information about potential side effects. CoDReS is more disease-driven since through its functional component it tests drug-targeted genes against a training set of important genes on IPF. Except from the FDA approved drugs for IPF (nintedanib and pirfenidone) we searched the European Medicines Agency and recent bibliography for other potential inhibitors of IPF (Table 8) in order to compare their chemical structures to our repurposed drugs.

**Table 8:** Other known potential inhibitors of IPF.

Drugs against IPF	Source
nintedanib	FDA approved
pirfenidone	FDA approved
1-(6-Benzothiazolylsulfonyl)−5-chloro-1H-indole-2-butanoic acid	European Medicines Agency
2-(2-Chlorophenyl)-4-[3-(dimethylamino)phenyl]-5-methyl-1H-pyrazolo[4,3-C]pyridine-3,6(2 H,5 H)-dione	European Medicines Agency
macitentan	European Medicines Agency
sildenafil	Treatment of idiopathic pulmonary fibrosis: a network meta-analysis^83^
omipalisib	Exploration of a potent PI3 kinase/mTOR inhibitor as a novel anti-fibrotic agent in IPF^13^
vorinostat	Drug Repurposing of Histone Deacetylase Inhibitors that Alleviate Neutrophilic Inflammation in Acute Lung Injury and Idiopathic Pulmonary Fibrosis via Inhibiting Leukotriene A4 Hydrolase and Blocking LTB4 Biosynthesis^15^
4-(dimethylamino)-N-[7-(hydroxyamino)-7-oxoheptyl]benzamide	Drug Repurposing of Histone Deacetylase Inhibitors that Alleviate Neutrophilic Inflammation in Acute Lung Injury and Idiopathic Pulmonary Fibrosis via Inhibiting Leukotriene A4 Hydrolase and Blocking LTB4 Biosynthesis^15^

**Figure 7:**
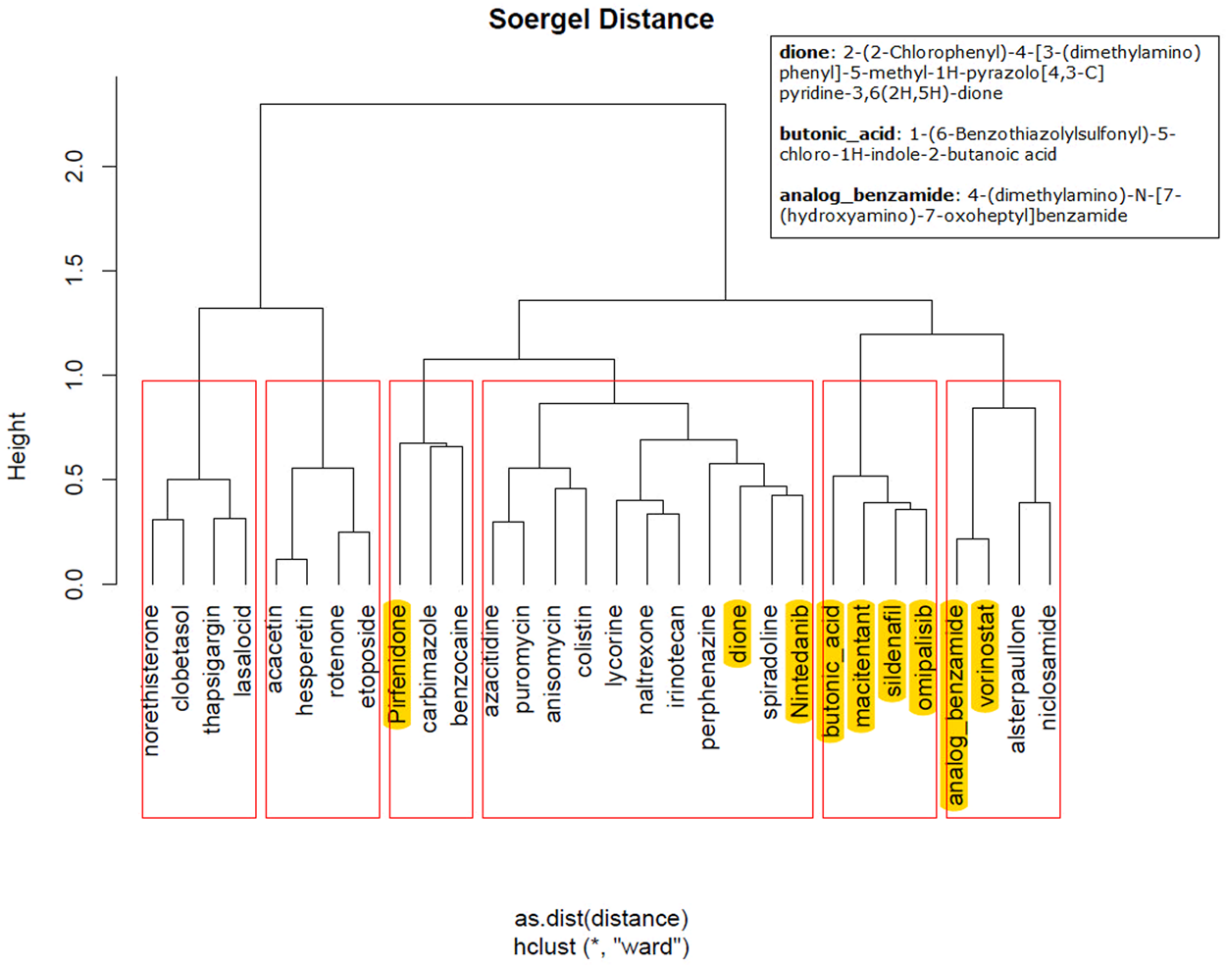
Hierarchical clustering of repurposed drugs and other known potential IPF inhibitors.

We used a structural similarity clustering in order to discuss the differences between the repurposed drugs’ ranking given by the simple inhibition score and the final CoDReS re-ranking score. The hierarchical clustering was calculated using ChemBioServer with Ward linkage, Soergel distance and a clustering threshold of 1 (Fig. 7).

The substances with the closest structural similarity to the known potential IPF inhibitors (highlighted in yellow), which are also clustered with them, are niclosamide, alsterpaullone, carbimazole, benzocaine, spiradoline and perphenazine. The CoDReS re-ranked drugs appear to have increased structural similarities with the known potential inhibitors of IPF rather than the ones received by inhibition score only. With the exception of benzocaine which drops one or two ranks compared to the inhibition score list for every experiment, each of the five other drugs gains 1 to 9 positions compared to the initial ranking (based on the simple inhibition score) per experiment.

### Pathways and targets

**Figure 8:**
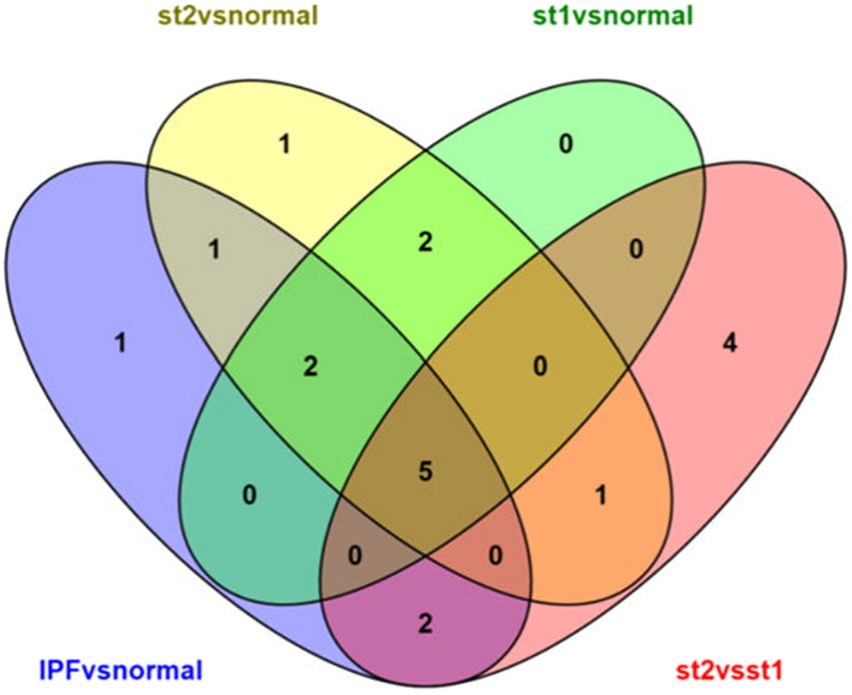
Venn between important pathways of each stage of the disease.

A Venn depiction of the intersection between the common pathways of all stages is shown in Fig. 8. Cell communication, ecm receptor interaction, focal adhesion, cytokine cytokine receptor interaction and colorectal cancer are the five pathways that were found in all four experiments. Functions regulated in fibroblasts that directly influence fibrogenesis are known to enhance or inhibit extracellular matrix (ECM) protein synthesis^50^. Recent findings suggest that focal adhesion kinase (FAK) plays a key role in development of fibrotic disorders, and it appears to be an attractive target for antifibrotic therapy^51^. In another study, FAK expression and activity were up-regulated in fibroblast foci and remodelled vessels from lung fibrosis patients. Their results implicate FAK as a central mediator of fibrogenesis, and highlight this kinase as a potential therapeutic target in fibrotic diseases^52^. Numerous cytokines have been implicated in the pathogenesis of lung fibrosis, including transforming growth factor-β (TGF-β), tumour necrosis factor-α (TNF-α), platelet- derived growth factor (PDGF), insulin-like growth factor-1 (IGF-1), endothelin-1 (ET-1) and the interleukins, (IL)-1 and IL-8. TGF-β, TNF-α and ET-1 are the strongest profibrotic competitors. On the other hand, there is evidence from animal and human studies that interferon-γ, which is also a cytokine may have antifibrotic effect^53^. As far as cancer pathways are concerned, IPF is known to have many similarities in its molecular mechanisms as cancer diseases. There are many links between IPF and cancer as mentioned in the study of Vancheri *et al*.^54^. All the pathways found for each in *silico* experiment study are shown in Table 9.

**Table 9:** Pathway results for each experiment.

IPF vs normal	Stage 2 vs Normal	Stage 1 vs Normal	Stage 2 vs Stage 1
cell communication	cell communication	cell communication	cytokine cytokine receptor interaction
ecm receptor interaction	ecm receptor interaction	ecm receptor interaction	hematopoietic cell lineage
focal adhesion	focal adhesion	focal adhesion	mapk signaling pathway
metabolism of xenobiotics by cytochrome p450	arachidonic acid metabolism	cytokine cytokine receptor interaction	long term depression
cytokine cytokine receptor interaction	metabolism of xenobiotics by cytochrome p450	arachidonic acid metabolism	cell cycle
complement and coagulation cascades	cytokine cytokine receptor interaction	metabolism of xenobiotics by cytochrome p450	cell communication
long term depression	bladder cancer	bladder cancer	prion disease
calcium signaling pathway	complement and coagulation cascades	complement and coagulation cascades	small cell lung cancer
urea cycle and metabolism of amino groups	cell cycle	colorectal cancer	focal adhesion
colorectal cancer	urea cycle and metabolism of amino groups		natural killer cell mediated cytotoxicity
hematopoietic cell lineage	tryptophan metabolism		colorectal cancer
	colorectal cancer		ecm receptor interaction

There are many bibliographic mentions for most of the pathways found by our study in relation to IPF. In reference to complement and coagulation cascades the pathogenesis of idiopathic interstitial pneumonia (IIP) shows that among other potentially relevant differentially expressed transcripts are complement components (B factor, H factor-1, and complement factor H–related 3), members of the coagulation cascade (tissue factor pathway inhibitors 1 and 2)^55^. Studies have shown that patients might suffer from depression due to subjective breathlessness but is unrelated to the fatigue caused by the disease^56^. In another study, depression questionnaires show significantly different depression levels for patients with IPF compared to control samples although the scores do not indicate significant cases of anxiety or depression. Also, the depression scores are significantly correlated with the duration of the disease^57^. For the calcium signaling pathway, myofibroblasts, which are the source of fibrotic tissue, are differentiated by a TRPV4-mediated Ca^2+^ influx. The presence of extracellular calcium is required for a rise in intracellular calcium. Human lung fibroblasts showed an absolute requirement for extracellular calcium to differentiate into myofibroblasts^58^. Urea cycle and metabolism of amino groups have also been related to IPF before. Arginase, which converts l-arginine into l-ornithine and urea, plays an important role in the pathogenesis of IPF. Remarkable increases of pulmonary arginase activity have been observed in patients with IPF^59^. Also, some cytokines, for instance IL-4 or IL-13, which are implicated in various lung disorders, also behave as inducers of arginase. The levels of arginase are also connected with the progression of pulmonary fibrosis^60^. As far as hematopoietic cell lineage is concerned, it is mentioned that the expression of the hematopoietic progenitor cell marker Thy-1 by regenerating epithelial cells in idiopathic pulmonary fibrosis indicates the potential for hematopoietic cells to enter the damaged regions of lung parenchyma to attempt to heal the injury^61^. Leukotrienes (LTs) are pro-inflammatory and pro-fibrogenic mediators derived from the 5-lipoxygenase (5-LO) pathway of arachidonic acid metabolism. Wilborn *et al*. indicate in their study that the 5-LO pathway is constitutively activated in the lungs of patients with IPF^62^. During the cell division phase of the cell cycle telomere shortening might occur. Defects in telomere maintenance have been linked to epithelial cell senescence and an impaired response to epithelial injury. Tryptophan metabolism was returned as a unique pathway result in the stage 2 vs normal experiment. Patients with inflammatory lung disease had decreased tryptophan as mentioned in the study of Meyer *et al*.^63^. Mapk signaling pathway is present in the stage 2 vs stage 1 IPF experiment. MAP kinases (MAPk) (eg ERK, JUN and p38 MAPK) are widely known to be involved in the regulation of lung inflammation and injury^64–67^. Natural killer cell mediated cytotoxicity is also a pathway only presented in the stage 2 vs stage 1 experiment. Natural Killer (NK) cells are lymphocytes which can activate cytotoxicity receptors. NK cells may mediate a protective effect against fibrosis^68^. The Fas ligand is predominantly expressed in activated T lymphocytes and is one of the major effector molecules of cytotoxic T lymphocytes and natural killer cells. Results suggest that the Fas-Fas ligand pathway plays an essential role in the development of pulmonary fibrosis and that preventing this pathway could have therapeutic value in lung injury and fibrosis^69^. We searched the KEGG database for the lists of genes contained in each pathway of our results. We checked which of our drugs’ targeted genes exist in the gene lists of the pathways. Nintedanib targets 7 out of the 13 total pathways being the best drug competitor as far as pathways are concerned. Table 10 shows the nine pathways that are targeted by the drugs of our study plus nintedanib and pirfenidone.

**Table 10:** Drugs targeting the most important pathways.

Focal adhesion	Cytokine cytokine receptor interaction	Colorectal cancer	Metabolism of xenobiotics by cytochrome p450	Arachidonic acid metabolism	Bladder cancer	Long term depression	Hematopoietic cell lineage	Cell cycle
nintedanib	naltrexone	alsterpaullone	clobetasol	benzocaine	nintedanib	nintedanib	naltrexone	nintedanib
alsterpaullone	nintedanib	nintedanib	acacetin	etoposide			nintedanib	alsterpaullone
			hesperetin	norethisterone				
			etoposide	perphenazine				
			perphenazine	irinotecan				
			irinotecan					
			benzocaine					
			norethisterone					
			niclosamide					

### Validation for the candidate genes

In the final stage of our research we tried to compare some of our results with other studies. We compared our gene lists with the gene lists proposed by the DLCO experiments (dataset GSE33566) for each stage as shown in the study of Yang *et al*.^70^. DLCO proposed genes are shown on Table 11 where common genes between DLCO and our lists from the respective stages of the disease are highlighted in bold.

**Table 11:** Important genes as shown in the study of Yang *et al*. and overlap with the genes from our experiments per IPF stage.

Mild DLCO ≥ 65% (stage 1 vs normal)	Severe DLCO ≤ 35% (stage 2 vs normal)	Severe vs mild DLCO (stage 2 vs stage 1)
CEACAM4	**IL1R2**	CAMP
**IL1R2**	DEFA3	CEACAM6
FCN1	**OLFM4**	**CTSG**
GRN	MMP9	**DEFA3**
PTGIR	GRB10	**DEFA4**
HLA-B	DEFA4	OLFM4
DYSF	**LTF**	HLTF
LILRB3	RAB8A	**PACSIN1**
TALDO1	CTSG	FLJ11710
**CXCR2**	CAMP	GABBR1
**FKBP5**	CEACAMP8	IGHM
SORL1	VSIG4	
IMPDH1	PGLYRP1	
DAPK2	**FKBP5**	
CA4	LOC151438	
MMP9	ECHDC3	
PSAP	LOC100130890	
TUBA3D	PRSS36	
RPL24	MCAT	
GPR78	IGHM	

We searched the common genes in bibliography and found out that most of them have already been related to fibrotic diseases or to the pathways mentioned in Table 9. Information about these genes was extracted from GeneCards^71^. OLFM4, which is found in stage 2 vs normal and stage 2 vs stage 1 experiments is known to be expressed in inflamed colonic epithelium, promotes tumour growth and facilitates cell adhesion. LTF regulates cellular growth and has a protective role against cancer. Its protein, lactoferin is known to be related with cystic fibrosis. LTF is also known to stimulate cell migration and proliferation via VEGFA gene, whilst nintedanib targets the receptors for VEGFA, VEGFR1 and VEGFR2. IL1R2 codes a cytokine receptor and the cytokine-cytokine receptor interaction pathway is found to be important in all the stages of IPF. Mapk signaling pathway is also related to IL1R2. FKBP5 is known to be related with myelofibrosis, another type of fibrotic disease in which the bone marrow is replaced by fibrotic tissue. DAPK2 is a kinase that is involved in cellular signalling pathways that are related to the cell cycle and specifically to cell apoptosis. It is also related with cancer pathways, such as bladder cancer. CA4 is a zinc metalloenzyme of the carbonic anhydrases family that participates in respiration and encodes a specific membrane isozyme which can be found on the luminal surfaces of pulmonary capillaries. CXCR2 encodes a protein which functions as a receptor for IL8. Studies have shown that IL8 might be a potential biomarker for IPF^72^. MMP9 is involved is the breakdown of the extracellular matrix (ECM). Ecm receptor interaction is a pathway that has been deemed important in every stage of IPF from our study. MMP9 has already been linked to IPF in various studies^73–75^. CTSG (cathepsin G) is a protein that participates in connective tissue remodelling of inflammation areas. Like MMP9, this gene is also related to the degradation of ecm pathway. CTSG is also related to cystic fibrosis^76^. DEFA3 and DEFA4 are peptides called defensins which can be found on various epithelia of mucosal surfaces including the respiratory tract. Bibliographically, PACSIN1 does not seem to be linked to fibrotic diseases as far as pathways and mechanisms are involved.

### Fibrosis and microRNAs

For the last part of our study, we searched for microRNAs that are related to fibrotic diseases in HMDD v2.0 (Human microRNA Disease Database version 2.0)^77,78^ (Table 12). We then found their gene targets (3384 genes) in mirTarBase^79,80^ and compared them with the gene targets of the repurposed drugs from our study. The results are presented in Table 13, where microRNAs in bold inhibit IPF and microRNAs in italics induce IPF.

**Table 12:** Fibrosis-related microRNAs as found in HMDD v2.0.

microRNA	Disease
hsa-mir-155	CysticFibrosis
hsa-mir-208a	EndomyocardialFibrosis
hsa-mir-25	EndomyocardialFibrosis
hsa-mir-29a	EndomyocardialFibrosis
hsa-mir-101-1	EndomyocardialFibrosis
hsa-mir-101-2	EndomyocardialFibrosis
hsa-mir-192	Fibrosis
hsa-mir-21	Fibrosis, PulmonaryFibrosis
hsa-mir-29b-1	Fibrosis
hsa-mir-29b-2	Fibrosis
hsa-mir-29c	Fibrosis
hsa-mir-31	PulmonaryFibrosis

**Table 13:** Common gene targets between microRNAs and repurposed drugs. miRNAs in bold inhibit IPF and miRNAs in italics induce IPF.

Common targets	Drug			micro RNA		
PLK1	nintedanib			*hsa-miR-155-5p*		
CDK5	alsterpaullone			*hsa-miR-155-5p*		
DNMT1	azacitidine			*hsa-miR-155-5p*	**hsa-miR-29a-3p**	
FLT1	nintedanib			*hsa-miR-155-5p*		
EGFR	nintedanib			*hsa-miR-155-5p*	*hsa-miR-21-5p*	
CDK2	nintedanib	alsterpaullone		*hsa-miR-155-5p*	**hsa-miR-29a-3p**	
CDK4	nintedanib			*hsa-miR-155-5p*	**hsa-miR-25-3p**	**hsa-miR-29a-3p**
GSK3B	alsterpaullone			*hsa-miR-155-5p*		
CYP1A1	clobetasol	acacetin	hesperetin	*hsa-miR-155-5p*		
CYP1B1	acacetin	hesperetin		**hsa-miR-208a-5p**		
TP53	nintedanib			**hsa-miR-25-3p**		
ERBB2	nintedanib			**hsa-miR-25-3p**	*hsa-miR-21-5p*	
CYP2C19	norethisterone	perphenazine		**hsa-miR-25-3p**		
PDGFRB	nintedanib			**hsa-miR-29a-3p**	**hsa-miR-29c-3p**	
ABCG2	irinotecan			*hsa-miR-192-5p*		
ABCC3	etoposide			*hsa-miR-192-5p*		
TOP1	irinotecan	etoposide		*hsa-miR-192-5p*		
IGF1R	nintedanib			*hsa-miR-192-5p*	*hsa-miR-21-5p*	
SOAT1	hesperetin			*hsa-miR-192-5p*		
CALM1	perphenazine			*hsa-miR-21-3p*		
TOP2A	etoposide			*hsa-miR-21-5p*		
SRC	nintedanib			**hsa-miR-31-5p**		

**Figure 9:**
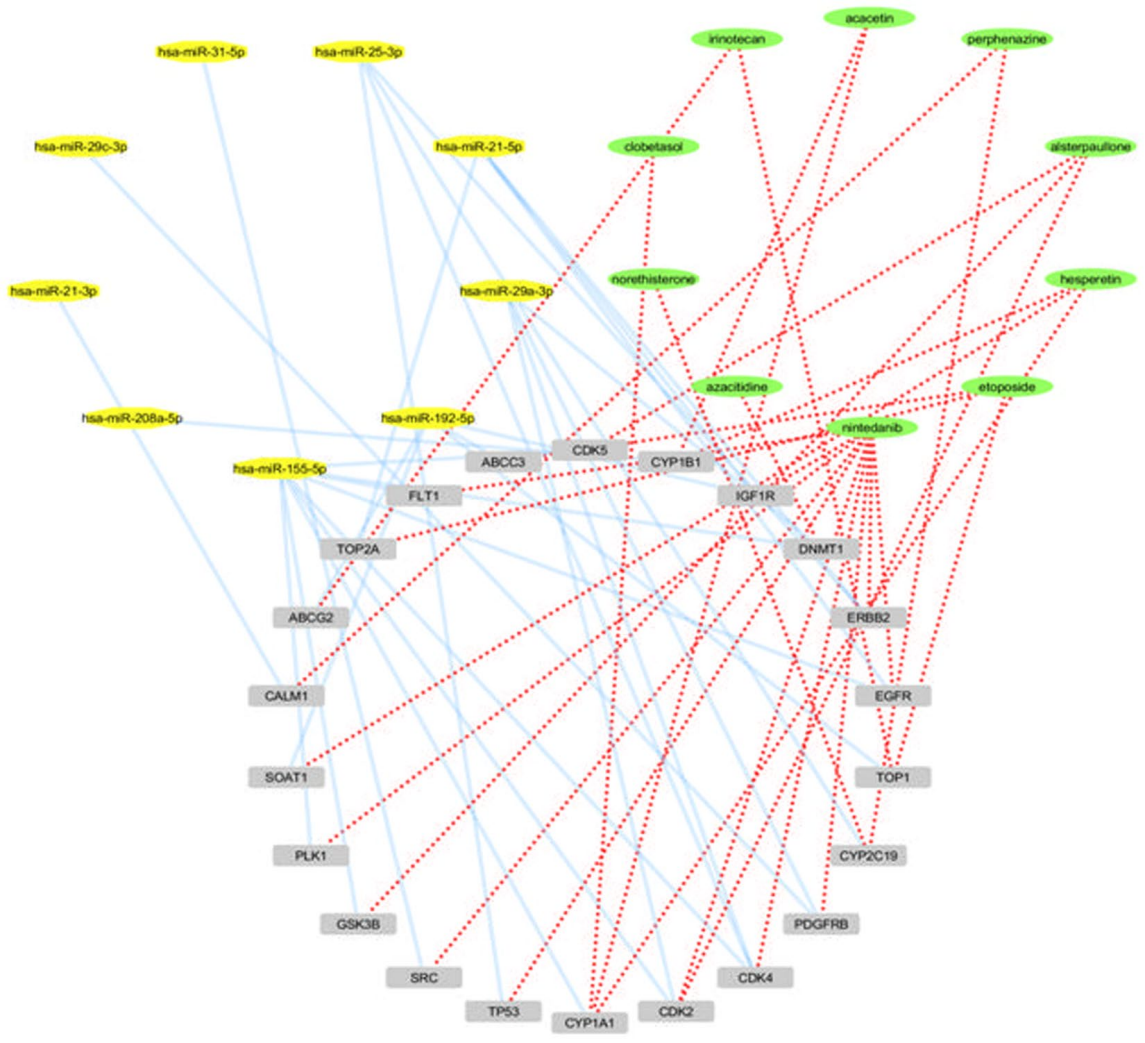
Network with IPF related common gene targets between microRNAs and repurposed drugs.

To further examine the results, we constructed a super network that combines the related microRNAs with IPF and the repurposed drugs that share common target genes (Fig. 9).

**Figure 10:**
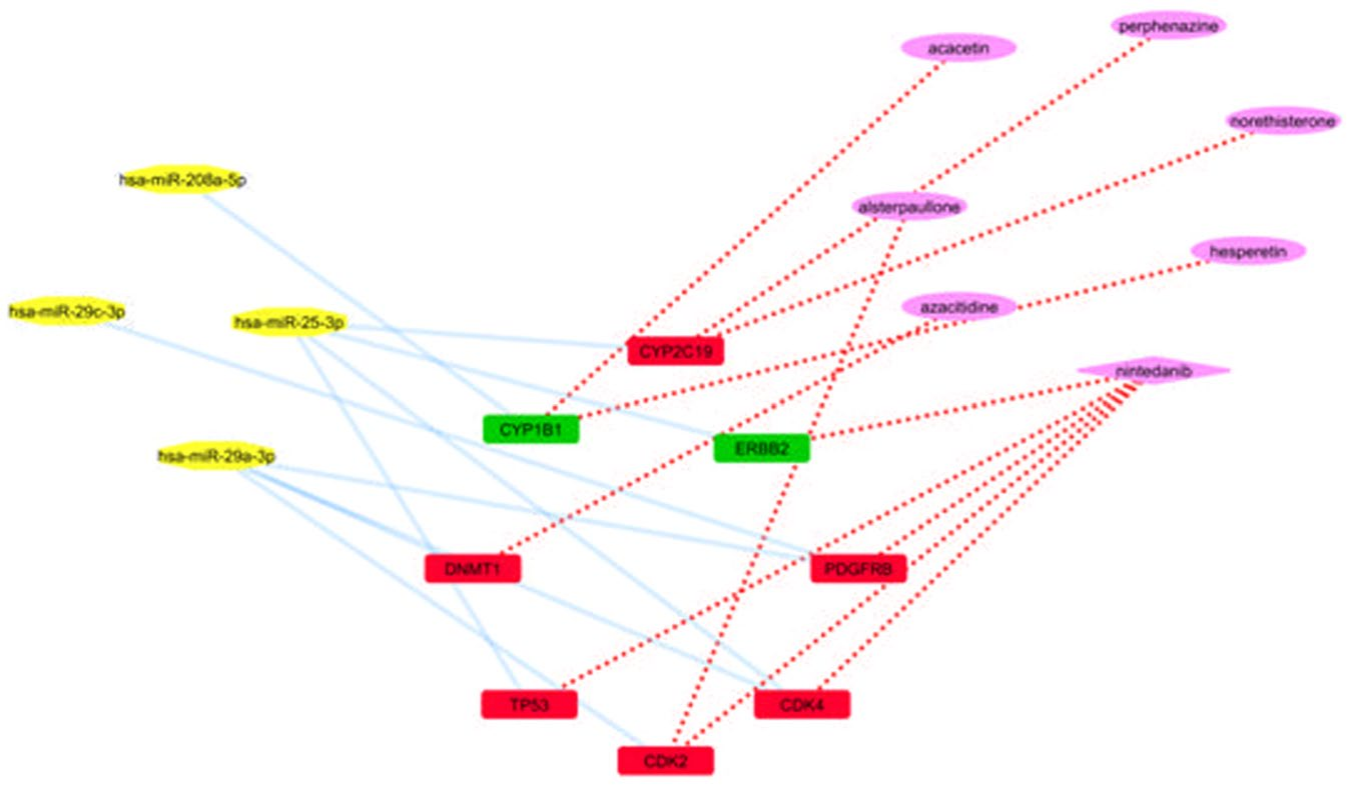
microRNAs and repurposed inhibitors that suppress IPF.

We examine only the microRNAs that were found to suppress IPF with their corresponding gene targets and repurposed drugs, as stated in HMDD Database. As microRNAs usually induce gene silencing by binding to target sites found within the 3′UTR of the targeted mRNA, we examine the inhibitor-microRNAs that target genes which were found to be overexpressed in our analysis. Finally, we concluded to two microRNAs that target two upregulated genes respectively (Fig. 10).

The first microRNA (hsa-miR-25-3p) targets ERBB2 gene that was found from our analysis to be up regulated in the IPF vs Normal experiments. This interaction is backed up by strong experimental evidence on MirTargetLink^81^ (https://ccb-web.cs.uni-saarland.de/mirtargetlink/index.php). ERBB2 is a member of ErbB family of receptors. It has been reported that the expression levels of the epidermal growth factor receptor (EGF-R) increases in IPF^82^. ERBB2 is also a target gene of the FDA approved drug Nintedanib, which inhibits it.

The second microRNA is hsa-miR-208a-5p. This miRNA targets CYP1B1 gene that was found from our analysis with Netwalker, to be up regulated in IPF stage 2 vs normal. This association is backed up by weak experimental evidence on MirTargetLink (https://ccb-web.cs.uni-saarland.de/mirtargetlink/network.php?type=miRNA&qval=hsa-miR-208a-5p). CYP1B1 is also a target gene of two repurposed drugs, Perphenazine and Norethisterone. Perphenazine is a typical antipsychotic drug. It is used to treat psychosis (e.g. in people with schizophrenia and the manic phases of bipolar disorder). On the other hand Norethisterone is a steroid alprogestin with additional weak androgenic and estrogenic activity that is used as a hormonal contraceptive in combined oral contraceptive pills and progestogen-only pills as well as alone or in combination with an estrogen for the treatment of gynecological disorders or hormone replacement therapy for menopause.

Finally, the total action of the two microRNAs (hsa-miR-25-3p and has-miR-208a-5p) must be further investigated, since they have a functional purpose as potential therapeutic drugs for IPF.

## Discussion

In the present work, we present a bioinformatics pipeline that analyses IPF gene expression data on early and advanced stages of the disease deriving candidate repurposed drugs for IPF and specifically for each stage respectively. The resulting repurposed drugs are re-ranked *in silico* using our own composite drug repurposing score CoDReS. The resulted drugs have been compared structurally and biologically with the FDA approved drugs already used in IPF, as well as with other known potential inhibitors of IPF. Moreover, we looked for natural compounds with structural similarity to the resulted repurposed drugs for IPF. Finally, we searched for microRNAs that are related to fibrotic diseases and we compared their gene targets with the gene targets of the repurposed drugs from our study. During this assessment, we concluded to gene lists (Supplementary Table 2) that provide strong evidence of relation to IPF because they appear in at least two out of three experiments (different datasets) of the same stage of the disease. Moreover, we cross validated our gene results with the results from Yang *et al*. study and found 4/21 common genes in the stage 2 vs normal, 6/21 common genes from the stage 1 vs normal and 5/11 common genes at the stage 2 vs stage 1 experiments. Nearly all of these bold lettered genes of Table 11 have been previously linked to fibrotic or pulmonary diseases or to pathways that are related to IPF except for PACSIN1, which might offer new insights for the treatment of IPF.

Furthermore, our analysis concluded to drugs (Table 2) that provide strong connection with IPF since they have been discovered by at least two out of three drug repurposing tools, with a given inhibiting score less than −0.5 against IPF. We then used our scoring formula in order to re-rank all the potential IPF inhibiting drugs, that passed the aforementioned rules, based on their inhibiting, functional, structural and side effect scores for each stage of the disease. Of all the resulting drugs and through bibliographic research regarding their connection to IPF we propose certain drugs as potential competitors against IPF. Niclosamide has been highly ranked by CoDReS for every stage of IPF, it is in the same structural cluster with other potential IPF inhibitors and has also been mentioned to reverse fibrosis on the skin and lungs of mice that had Systemic Sclerosis with pulmonary fibrosis. Due to its safety profile, niclosamide can be considered for use in clinical trials against IPF. Anisomycin and Puromycin, natural compounds that both derive from the Genus of streptomyces, seem to have potential against IPF; Anisomycin is known to inhibit the induction of ER stress and Puromycin targets inflammatory cells. Etoposide and Irinotecan are used for cancer treatment and IPF is known to have similar mechanisms with various types of cancer.

Most of the specific mechanisms (Cell communication, ecm receptor interaction, focal adhesion, cytokine-cytokine receptor interaction and colorectal cancer) that were found in all experiments, are related to fibrotic or pulmonary diseases or to other pathways that are related to IPF. This is a means to test the validity of our methods but at the same time it points out more pathways, potentially involved in IPF. As seen in Table 10, most of the aforementioned pathways are targeted by repurposed drugs from our study. This strengthens our claims for new potential repurposed drug inhibitors for IPF.

In the case of microRNAs, hsa-miR-25-3p and hsa-miR-208a-5p can inhibit genes that the FDA approved drug nintedanib targets. This finding provides an insight for a new microRNA-based treatment approach.

Finally, the action of the remaining mechanisms and drugs that were found from our analysis, may be further investigated, since they have been derived from significantly relevant genes related to IPF stages.

## Electronic supplementary material

Supplementary Information
