# Supplementary material for: Drug repurposing in idiopathic pulmonary fibrosis filtered by a bioinformatics-derived composite score
Source: Sci Rep. 2017 Oct 3;7:12569. doi: 10.1038/s41598-017-12849-8 (PMC5626774; doi:10.1038/s41598-017-12849-8)

Drug repurposing in idiopathic pulmonary fibrosis filtered by a bioinformatics-derived composite score

E Karatzas[1], M Bourdakou[2,4], G Kolios[3] and G Spyrou[4,*]

[1] Department of Informatics and Telecommunications, University of Athens, 15784 Ilissia Athens, Greece.

[2] Center of Systems Biology, Biomedical Research Foundation, Academy of Athens, Soranou Ephessiou 4, 115 27 Athens, Greece.

[3] Laboratory of Pharmacology, Department of Medicine, Democritus University of Thrace, Greece

[4] Bioinformatics ERA Chair, The Cyprus Institute of Neurology and Genetics, Cyprus

[*] Corresponding author. George M. Spyrou, Tel.: +357 22 392852; E-mail: georges@cing.ac.cy

Supplementary Table 1. The derived gene lists per dataset from Limma's statistical analysis and from NetWalker's random walk approach.

IPF vs normal											
GSE10667				GSE24206				GSE44723			
Limma		NetWalker		Limma		NetWalker		Limma		NetWalker	
up	down	up	down	up	down	up	down	up	down	up	down
MMP1	ITLN2	KRT6B	IL6	BPIFB1	IL1R2	MMP1	IL6	DSG2	SLITRK6	MGP	CCNB1
COL17A1	CHIAP2	KRT15	SH3GL2	MMP7	S100A12	MMP7	ZBTB16	DSP	ARHGAP28	BMP2	RUNX2
KRT6A	RTKN2	KRT14	IL6ST	CXCL14	ARG1	MDK	FOS	MFAP5	IQGAP2	CPE	PRKAR2B
COMP	SERTM1	KRT5	SOSTDC1	SFRP2	IL6	PTPRZ1	CEBPD	STK26	CCDC102B	PMCH	PBK
SERPINB3	AGBL1	UGT1A6	BMP2	SERPIND1	DEFA1B	TSPAN1	IL6R	GSTT1	MASP1	FGF1	SLC38A4
BPIFB1	KLRG2	CYP2F1	EDNRB	MSMB	MGAM	MS4A2	STAT3	SLC39A8	THRB	SYT1	KYNU
SPP1	SLC6A4	GSTA2	CAV1	MMP1	PTX3	FCER1A	LIF	PTHLH	KYNU	ARAP2	PRC1
KRT5	HTR3C	GSTA1	EDN1	COL1A1	IL18RAP	SPP1	LIFR	MFAP3L	CLDN23	ARHGDIB	CENPE
IGFL2	NECAB1	GSTA5	IL6R	MUC5B	PROK2	IGF1	IL6ST	EMB	IL7	RHOH	KIF4A
BPIFA1	CA4	CHST6	COL6A6	KRT17	MT1M	CCL5	FOXO1	GCH1	MATN3	CD3D	MAOA
MMP10	CRTAC1	B3GNT3	COL4A3	SNTN	SLCO4A1	CXCL11	OSM	CCND2	WFDC1	ITK	AOX1
SERPINB4	SLCO1A2	EYA1	WNT7A	ASPN	BTNL9	CXCL10	MAFF	ARL4C	ROBO2	PTPRC	HNMT
UGT1A6	DPP6	SIX1	FZD5	HS6ST2	ADM	CXCL5	NFE2	APBB1IP	METTL7A	ARHGAP15	CCNB2
RPS4Y1	SLC5A9	TUBB2B	CTNND2	UBD	LOC100288	CXCL6	CSF3R	HECW2	SYTL2	FNBP1L	NEK2
PCSK1	ECEL1P2	TUBB3	PDZD2	COL15A1	AFF3	APLNR	PLA2G1B	KRT34	CES1	SLC6A15	BIRC5
GPR87	F11	KRT6C	CA4	S100A2	SOD2	CXCL12	SOCS3	BAALC	GPR126	SLC7A5	CENPF
TMPRSS4	GPR158	KRT17	CA2	LPPR4	IL18R1	UGT1A3	CXCR2	SYT1	COL3A1	SLC1A4	ARHGAP6
RPS4Y2	SOSTDC1	KRT6A	BMPER	COMP	IL1RL1	CYP2F1	CXCL3	GATA6	SCN4B	CDO1	CARD16
SAA4	HSD17B6	SRD5A2	CYP3A5	CRIP1	TTN	KRT5	IL8	OXTR	SLC38A4	FN1	SNCAIP
IL13RA2	HMGCS2	BAAT	CYP3A7	EPHA3	SAMSN1	TP63	ACSL1	FNDC1	MYOCD	TRIB3	PTN
PLEKHS1	RS1	PLA2G2A	CYP3A4	PROM2	SERPINA3	GSTA1	ACADL	KIF21A	SEPP1	APBB1IP	COL3A1
KRT6C	MYRF	UGT2B17	PLA2G1B	CTHRC1	ORM2	EPHX1	OLAH	PCDH10	HOPX	IL6	PCOLCE
CRLF1	C8B	IGHG1	MME	SCGB1A1	SOCS3	IGFBP4	HBEGF	CPE	MAP2	PTHLH	MATN2
KRT14	IL13	IGKC	SLCO1A2	KRT5	VNN2	IGFBP2	SOD2	DHRS9	EYA4	LCK	MATN3
GREM1	PLA2G1B	IGF1	SLC26A9	FAM81B	CXCR2	COL1A2	GPX3	ICAM1	TMEM155	ST6GAL1	MAP3K5
KLK12	AGER	MMP1	WNT3A	C9orf24	CLEC4D	COL1A1	CCL20	SULF1	VAT1L	FUCA1	KIF23
KRT17	CTNND2	GSTA3	SH3GL3	GSTA1	TIMP4	COL3A1	AGTR1	KRT19	RGS17	ARHGAP26	F3
MMP13	GRIA1	ALDH3A1	HLA-DQA1	PSD3	CXCL8	EYA2	ADORA3	EPHA5	AKR1C3	PTPRB	TFPI
MSMB	BTNL9	SPP1	PLA2G12B	SPATA18	RNASE2	SIX1	MXD1	PTPRR	TSLP	VAV3	NQO1
PLA2G2A	BDNF	COL1A1	CHRM3	CCDC113	SLC7A11	CXCL13	MYC	SLC1A4	SYTL5	PNMA2	SPAG5
SCG5	LINC00968	THBS2	AGTR2	KRT15	DUSP1	CCL19	KLF4	PTPRB	FAM162B	CYFIP2	CEBPD
MMP7	HHIP	COL3A1	SFTPA1	RSPH1	APOBEC3A	COL5A1	KLF6	TGFA	PLK2	CD55	SGOL2
AKR1B10	CLDN18	SLC27A2	SFTPA2	ZBBX	FMO5	ST6GALNA	ORM1	FGF1	TRPC6	PECAM1	SNCA
SCRG1	MT1E	HTR2A	ADCY8	CXCL13	FPR1	GALNT6	SERPINE1	KCNK6	FLRT3	DSP	NUF2
PODNL1	CCK	MAP1A	CXCL3	ITLN1	CHI3L2	CCND1	ORM2	FUCA1	ST6GALNA	SFRP4	FGF7
SAA1	CYP3A7	CYP2A6	VIPR1	SOX2	HAL	XPO1	S100A8	ANKH	ARHGAP6	FZD8	CDK1
CXCL6	CYP3A4	LTF	ADRB2	NTM	FPR2	UGT1A6	S100A9	ZPLD1	ACTC1	CCL20	TOP2A
LGALS7	AGTR2	CP	CHRM2	GPR87	FKBP5	KRT14	SLC2A14	SCG5	F2RL2	CXCR4	AMPH
LGALS7B	SGCG	MMP7	ADRB1	FNDC1	FAM107A	AGBL2	SLC2A3	NAP1L3	TRPA1	CD8A	CDKN3
LCN2	TNNC1	DST	CCR3	CAPS	ADORA3	PPBP	MGAM	KCNJ2	HGF	NTS	CXCL12
PSCA	APOH	COL17A1	APLN	MMP10	FOSB	TRBC1	HNRNPU	STC2	PTN	IKZF2	SST
PROM1	TINCR	RHOV	NOS1	TPPP3	ZNF385B	HLA-DOA	FUS	KCNC4	RUNX2	IKZF1	CDC25C
COL1A1	LGALS2	SRGAP3	TNNC1	ITGBL1	NFKBIZ	HLA-DQA1	CYP3A5	CHST11	ZNF608	ADRBK2	CHN1
S100A2	GPM6A	IVL	TNNT1	LRRC17	SDR16C5	SDC1	MAOA	CDO1	LRRN3	IGFBP3	CD44
SIX4	FGFBP2	KRT1	NOS2	COL14A1	SCN7A	AKAP14	CBS	PNMAL1	PIEZO2	PTPN22	HMMR
PIP	ZBED2	CCL7	FZD8	CHIT1	NR4A2	MYCBP	CTH	FAM171A1	LINC00619	IL8	ANGPT1
CTHRC1	SLC14A1	ACAN	DAO	PROM1	S100A8	FGF14	SLC6A14	GRIA3	LDB2	SPP1	TEK
COL10A1	LRRC36	NGEF	RASGRF1	UGT1A3	HIF3A	FGFR3	AOX1	SLC7A5	ANO4	PDGFA	GNAI1
TNS4	WNT3A	CCL13	GJB1	C20orf85	CCL20	DNM1	AASS	TRIB3	LRCH2	CCND2	CCNA2
ADAMTS16	CYP3A5	DLG2	COL4A4	COL1A2	HNRNPU	KIAA1377	SLC7A11	KCNN4	ANGPT1	CDK6	PRKAA2
SFRP2	ASPG	MMP13	SPOCK2	DYNLRB2	MAFF	KRT17	GFPT2	FNBP1L	HNMT	WT1	CDKN1C
GSTA2	RBP2	RIBC2	PLXNB3	LRRC46	PGC	KRT6A	ARG1	FN1	PSG1	PAPPA2	CEP55
MMP11	KCTD16	PAPPA	ARAP2	IL13RA2	PADI4	ROBO1	USP53	ATF3	FOXF2	UGCG	NDC80
MMP3	EFR3B	PNOC	TES	COL3A1	CCDC141	ALDH1A3	HAL	FZD8	PCDH18	RAG1	F2R
SAA2	CNTN6	CXCL6	RAMP2	GABBR2	CSRNP1	ALDH3A1	PFKFB3	GPC4	SESN3	RAG2	F2RL2
CYP24A1	LNX2	TP63	SLC6A14	IGFBP2	FAM65B	CTSD	JAK1	ST6GAL1	MME	NKD2	PTTG1
CILP	VIPR1	FBLN2	DLC1	SPP1	NLRP3	THBS2	CXCR4	ARHGAP26	LINC01140	TGFA	ALDH7A1
P4HA3	SEMA3E	MFAP5	PTGS2	FCER1A	PHACTR2	CPE	HIST1H2BC	SCD5	SOX6	CD3G	DCN

DDX3Y	PRX	FBN2	CAV3	SERPINB5	APOLD1	ROBO2	BCL10	HTR2A	MAOA	SH2D1A	TGFB2
VTCN1	ODAM	SAA2	ADAMTS1	C12orf75	CLEC4E	IGFBP7	BCL6	IGFBP3	ADAMTS5	HERPUD1	HSPA2
GSTA5	CACNA2D2	LAMA1	VEGFA	ROBO2	NAMPT	FOXA1	PTGS2	PECAM1	DLX2	CSGALNAC	CEP70
MMP16	TNNT1	MMP3	DDC	CFAP43	CD69	KRT15	PMAIP1	LRRN4CL	KCNE4	CHST11	KIF18A
OGDHL	ADRB1	SPOCK1	SOCS2	CDCA7	RGL4	PTPN13	TLR2	CYP4V2	GAS2L3	MYB	RGS5
KIF26B	ANGPTL7	CCR2	LIFR	MUC4	SIGLEC10	PDLIM4	TLR4	CDK6	FAM13A	ZAP70	TBXAS1
CHRDL2	NCKAP5	ITGA11	HLA-DRB5	PLEKHS1	MYC	TF	ADRA1A	UGCG	PTPLAD2	SPEN	PTGS1
POSTN	LOC10013	GGT5	CER1	DNAH12	PLA2G1B	CCL13	CH25H	ITPR2	MIR155HG	PTGS2	MYLK
HS6ST2	CSF3	FGF7	CA12	COL10A1	GPR97	HTRA1	LDLR	TMEM171	RNF182	TGM2	ACTC1
KRT6B	LEPREL1	COL7A1	NR0B2	PCDH7	GPIHBP1	ENAH	CEBPB	LINC01003	TMTC1	NOTCH1	NES
CP	KRT72	PSAT1	GRIA1	TP63	EMP1	EVL	RGS2	PSAT1	PTGR1	SLC3A2	COL14A1
COL3A1	FAM167A	SAA1	HOMER1	SLC28A3	SERPINE1	ITGA2	ALOX15B	EPHB1	TSPAN12	ICAM1	CLEC3B
CLCA2	EPB41L5	CXCL5	CXCL2	MGP	FCAR	MYB	ND6	TNFAIP3	HEY1	GHR	HGF
KRT15	FAM105A	IGHA1	CADM1	SCGB3A1	HSPA1B	EFNA5	CYCS	LRP8	LOC10050	PTPRR	C1R
IGF1	RGS9BP	FBN1	MPP3	CD24	ST6GALNA	EPHA3	SPAG9	STK17B	TJP2	CDH2	MASP1
SOWAHA	ARC	IGFBP2	NFKB2	SLITRK6	ATF3	CD4	MGST1	TPK1	VCAM1	COMP	IL7R
GSTA1	FIGF	CDH2	CEBPB	ST6GALNA	EGR1	SOS1	PTPN11	CPPED1	AOX1	GNAS	TSLP
HTR2A	LHFPL3-AS	BOC	FGA	CLCA2	SELL	VCAM1	MAP2K6	HERPUD1	SELENBP1	FGD4	KIF2C
DIO2	MS4A15	MMP2	KHDRBS2	ITGB8	FAM150B	ITGA9	IRS2	PROSER2	GPR85	CTH	ALDH1A1
TIMP4	COL4A3	LCN2	HMGCS2	KLHL13	EDNRB	TGFB2	CA4	TNFSF9	GBP2	BMP6	UBE2C
CDH3	GRM8	TIMP4	HMGCS1	CDH3	CBS	LTBP3	CA3	ZCCHC2	HR	NCOA3	CPS1
SLN	KIR2DS4	CYP19A1	GP1BA	PTGFRN	ORM1	HS6ST2	HMOX1	ZXDC	ANO3	BCAT1	PLA2G5
CXCL14	MYH2	VCAN	F11	C11orf80	TMEFF2	HS3ST1	TOP1	ANKRD44	UNC5C	SSR1	GPX3
SPRR1A	EMP2	AGT	LIF	SPAG6	MT1X	NUP210	INSIG1	SERPINE2	AMPH	RPS4Y1	GAB1
ATP12A	CPB2	IGFBP5	SHANK2	SCG5	CEBPD	CYP2S1	HMGCR	TIMP3	RCAN2	JUN	CDCA8
SERPINA5	BTNL3	COL5A2	BAIAP2	CHST9	C11orf96	ITGB4	MT2A	SLC20A1	ARHGAP20	PAWR	CHRM2
CHST6	PEBP4	TDO2	EDN3	MUC16	SLC2A14	CLCA2	BMX	BAG1	SSX2IP	HSPB8	PYGL
PTPRT	CLIC5	SLC16A10	SLC4A1	KIAA1377	ATP13A4-A	DDR1	S1PR1	SPEN	LPAR6	HSPB7	CASC5
TSPAN19	IL6	COL6A3	CDH10	APLNR	PKHD1L1	NME5	NEDD9	SLC3A2	NDNF	C3	KIF20A
TUBB3	FENDRR	THBS4	PTN	CXCL10	CA4	DYDC2	PTPN12	IGFBP7	PAQR5	CYCS	C5
SOX2	DAO	IGLV1-44	SLIT2	FAM216B	S100A9	GPX7	PDGFRA	IL17RA	DCN	ALDH5A1	S1PR3
EFNB3	SH3GL2	PCSK2	ZBTB16	PRSS12	CYP4F3	SLC6A1	USP9X	BCAT1	PIR	ABAT	ANGPT2
PIH1D3	EFCC1	SCG5	RGS9BP	ABCA13	SLC19A2	GABBR2	JUN	SH3BP2	TFPI	UBASH3A	MKI67
HCAR1	JPH1	PLA2G7	RGS9	MXRA5	TACC2	CYP4X1	ETS2	GNAS	TP53TG1	LCP2	THRB
CCNO	NCR1	ADORA3	NR3C1	ZMAT3	FIGF	LGALS3BP	IRAK3	DIEXF	PLN	ITPR2	PPARGC1A
GJB2	ANKRD1	SLC1A3	FUT1	CAPS2	SLC6A14	ITGA4	IRAK2	SSR1	GPR137C	AGTR1	FOXM1
ERICH3	APLN	PLXNA4	AK5	IQCA1	PEBP4	ABCC3	PELI1	HINT3	KIF23	ALDH1A2	PPAP2A
TMEM229	PPP4R4	DPYSL4	SPHK1	CAPSL	CTH	FABP6	ADM	ITGB1BP1	SAMD11	SLC16A10	PDE4B
SPOCK1	NXF3	COL1A2	CREB5	SLC4A11	MXD1	MSH2	ADRB1	LOC28581	SNCAIP	SLC38A1	ASL
DNAH3	CCR3	BBOX1	FOSB	CXCL12	OLAH	LDOC1	DUSP1	ERP44	TRABD2A	PRKCQ	ADARB1
ADAMTS14	DAPK2	CFB	CACNA2D2	ANO1	PIGA	BGN	SLIT2	MPC2	HTRA3	EPHB6	PTK2
PSAT1	NMUR1	C6	SLC5A9	TDO2	ERRFI1	BSG	CLASP1	ADK	PRSS12	EPHB1	NR4A2
CXCL13	SLC39A8	IGKV1-5	GBA3	VASH1	NFE2	ASPM	CHRM3	ABAT	RALGPS2	RASGRP2	DPT
F2RL2	LINC00312	S100P	TNNT2	CCDC78	LDLR	MUC16	PTX3	NTNG2	TMEM220	PTPRCAP	COL4A1
RARRES1	AATK	S100A2	EIF5	VSIG1	NFIL3	MSLN	FGF2	SLC38A1	WDR35	SLA	TRIP13
CDH2	FOSB	KRT81	EIF4E	LRRN1	MMP8	CCL21	FLT1	PPP3CC	FMO3	CER1	SELENBP1
RAB3C	OLFML2A	UCHL1	SULT1A2	TMEM231	ADAMTS1	TOP2A	CXCR1	NIPA1	TNC	PLAT	DPYSL3
ACAN	FLJ34503	CCNA2	DNM3	TMEM190	RGS2	ITGB8	ETS1	GSAP	LOC10050	DPP4	SEMA3A
THBS2	GALNT13	CCNA1	PDGFB	WDR66	BTNL8	TGFB1	APOA2	SLC19A2	NES	COL1A1	TPX2
FAM92B	ESM1	BDKRB2	CAV2	SULF1	CSF3R	SFRP4	IL1B	IL17RD	RASL12	SH3BP2	CENPA
CHST9	MYZAP	ALDH1A3	S1PR1	CNTN3	MAOA	FZD3	AGER	ASB1	NQO1	ARHGAP9	ACTA2
PCDH7	LAMP3	GPT2	ST6GALNA	PDLIM4	SLC2A3	IL2RG	S100A12	RBM8A	SHANK2	FGF9	SPC25
C6	RXFP1	MAP1B	HAS1	MNS1	PTPRB	COL4A5	SLCO4A1	ATP13A3	PRKAA2	SOSTDC1	MAD2L1
IGHG1	KHDRBS2	PLN	HAS3	SCARA3	AKAP12	RGS5	SLC26A2	F11R	XPNPEP3	IGFBP5	LPAR1
MUCL1	IGFALS	SLN	HDC	TRIM29	CMTM2	GNAI1	PDE4B	FAM13B	TBX2	CBLB	HTR2B
PCP4	KIR2DS2	ADCY2	CD274	ADAM12	FLT1	MFAP2	RAPGEF4	ZMYM5	FAM49A	BMPER	LDB2
HSPA4L	CLIC3	TTR	TIMP3	NREP	EPB41L5	ADRA2A	SF1	TLE4	LXN	NGEF	LMO4
TDO2	GPIHBP1	PHYHIP	CEBPD	EGFEM1P	SLCO4C1	MMP2	CYP4F3	ACOT4	RBMS3	F11R	MET
IGLL5	SFTPA2	EFNB3	EPAS1	CCL18	CCDC71L	TCF7L2	LTB4R	CUX1	LPAR1	THBS1	ITGA1
RHOV	LINC00165	RHBDL2	PVRL3	CCDC170	CCK	KRT19	SOCS2	MINOS1	CCBE1	TUBA4A	RACGAP1
CCNA1	MATN3	FABP6	SLC22A3	POSTN	CD274	LEF1	PDE4D	THBS1	ANGPTL2	RAC2	COL4A2
GALNT15	DHRS2	ALOX15	SSTR1	DCLK1	CD177	KCNMA1	TSC22D3	BMP2K	S1PR3	SLA2	LRRK2
FCRL5	ADRB2	FLNC	NOG	GPX8	FOS	PTK2	IL1R2	C1orf198	TGFB2	SCD5	NOV
FAT2	ZNF385B	TF	IL20RA	SIX1	LOC10028	SPAG16	IL1RAP	NPEPL1	F2R	RBBP4	DLL1

RRM2	CD160	AK7	ACE	ECM2	CCDC85A	SPAG6	NFKBIZ	SLC20A2	TBX5-AS1	BCL11B	LAMC1
BAAT	PARD6B	RRM2	GRID2	C7orf57	GPR4	EPHX2	THBS1	GRAMD1B	CD44	BCL2	LAMB1
C9orf135	DDC	COL5A1	USHBP1	THY1	LOC10051	ABCC5	TFPI	SRD5A3	NRGN	WARS	BDKRB2
DPEP1	FAM189A2	DDX3Y	FAM107A	GREM1	GALNT15	PDE1A	NFIL3	MEF2A	ZNF395	PRKCB	CYB5R2
FAM150A	CADM1	MFAP2	STAT3	PPAP2C	BCL10	CXCL9	CREM	SRSF8	CREB5	GAP43	LAMA3
HES2	CHRM3	PYCR1	OCLN	SCN4B	ZBTB16	COL7A1	INMT	UBL3	CEP70	GRIA3	SPC24
NGFR	KIR3DL2	P4HA3	RAMP3	FRZB	SIK1	FABP4	P2RY1	PAWR	NUDT7	CBS	HEY1
FAM83D	S100A3	P2RY6	KLF6	C2orf40	FIBIN	PDE7B	IER3	SQLE	STOM	ABL2	SMAD3
ISM1	GGTLC1	ABCC5	SDC4	MS4A2	MYLIP	SDC3	MCL1	HCG11	PPAP2A	RAMP1	PRKACB
TTR	SH2D1B	PDE1A	PTPRB	DIO2	EMR2	BCL11A	PTPRG	WARS	MMD	DOCK8	AURKA
ERICH5	BEX1	TEKT4	TEK	PLXDC1	HHIP	MTA1	OSMR	USP36	ZNF300	RHOJ	PLK4
FAM216B	STC1	CDT1	WIF1	VSNL1	STC1	SFRP2	GRIA1	SLC7A6	PRDM6	INPP5D	SMC4
SIX1	EDNRB	PROC	PLK3	CCL5	CXCL3	GUCY1A3	FASN	PLD6	SNED1	PLCL1	SOCS3
TMEM45A	CCDC85A	SERPINA5	FOSL1	ALDH1A3	STC2	AKR7A2	ARHGEF7	CEBPG	NEURL1B	CYP1B1	MOXD1
B3GNT3	ANKRD29	FBLN1	MYH6	TRBC1	TBX5	ALOX15	NPR1	ITPR3	HLA-DMB	GSTT1	ROBO1
TUBB2B	HPGD	AGBL2	FGG	CDHR3	GPX3	SORD	B2M	CNPPD1	RGMB	FYN	ACACB
CFAP53	STXBP6	KCNMA1	GBX2	GXYLT2	PTPRG	LCN2	LCP2	ATF5	ECHDC3	HMHA1	DNM1
MRAP	CHIA	CXCL13	CACNG4	AKAP14	FFAR2	ROGDI	JUNB	ZNF652	PLA2G5	ARHGEF3	ROBO2
DYDC2	PRTG	CCL21	SLC2A3	CCDC34	INSIG1	MAD2L1	BTG1	SERP1	GSTA4	ADK	BUB1
EYA1	RND1	ADH4	CD244	CP	MAP3K8	UBD	ATF3	POLR1B	EREG	ADA	SNAP25
EFCAB10	KLRF1	CCL19	SH2D1B	IGFBP4	CLDN18	KLHL13	RPS6KA2	ABL2	DENND2A	CD59	WISP1
IGHV3-30	FAM189A1	CXCR5	ARHGEF4	MEOX1	LMCD1	SIX4	STRAP	KLHL21	ALDH6A1	CMPK2	TK1
KIF20A	ACADL	GNAO1	TAT	CYP2F1	C10orf54	SMAD1	ADRB2	GDPD1	TXNIP	ENPP1	PLN
IGHA1	SFTPD	DNM1	CSF3R	CIRBP	OSM	ZNF521	TPM3	C10orf85	C1RL	DNMBP	EDNRB
SCFV	C21orf90	SNAP25	STAT1	IGDCC4	PHACTR1	COL5A2	TTN	TRIM35	EPB41L2	PIK3R5	KIAA1377
LGI2	SFTPA1	CPT1C	C8A	GOLM1	STARD13-A	GGT6	CD44	SIPA1L1	OLFML1	HES1	BCL3
STEAP1	HLA-DQA1	CENPF	C8B	PTGDS	ACADL	PLCB4	FADS1	HIGD1A	TP53I11	SLC7A6	PIR
MUC16	TMSB15A	ABCC3	FOXA2	MS4A8	ETNK1	PPAP2C	TNFAIP3	MXD1	ADAMTS8	SPN	SMAD6

IPF stage 2 vs normal											
GSE10667 - acute				GSE24206 - advanced				GSE44723 - rapid			
Limma		NetWalker		Limma		NetWalker		Limma		NetWalker	
up	down	up	down	up	down	up	down	up	down	up	down
SERPINB3	ITLN2	FGF7	IL6	BPIFB1	IL1R2	MMP1	FOS	STK26	SLITRK6	FGF1	DLL1
SERPINB4	RTKN2	COL1A1	SH3GL2	MSMB	DEFA1B	MMP7	IL6	IRX3	KYNU	SYT1	NOV
BPIFA1	CHIAP2	COL3A1	GJB1	CXCL14	S100A12	SPP1	CEBPD	CMPK2	TRPA1	CD3G	SNCAIP
KRT6A	AGBL1	GSTA2	CAV1	MMP7	BTNL9	KRT5	IL6R	SLC39A8	CCDC102B	CD3D	PTN
MSMB	FOSB	GSTA1	CTNND2	MUC5B	AFF3	DST	IER3	EMB	VWA5A	ITK	F3
COMP	CRTAC1	GSTA5	PDZD2	MMP1	ARG1	TP63	MCL1	CRNDE	WFDC1	CD8A	TFPI
MMP1	KLRG2	ADORA3	RSPO1	SNTN	IL18RAP	MDK	SLIT2	HPGD	VAT1L	LCK	CDKN1C
BPIFB1	LGALS2	CXCL12	DKK1	SFRP2	PTX3	TSPAN1	CLASP1	PNMA2	TSPAN12	PTPRC	NR4A2
SAA4	LHFPL3-AS	CXCL6	GSTT1	SERPIND1	IL6	PTPRZ1	THBS1	ARL4C	ARHGAP28	RHOH	SLC38A4
SAA1	SERTM1	TDO2	CYP3A4	KRT17	MGAM	EPHX1	IL1R2	SLAIN1	ROBO2	ARHGAP15	KYNU
SAA2	NECAB1	SLC16A10	LDLR	C9orf24	PROK2	CYP2F1	IL1RAP	GCH1	AKR1C1	VAV3	SNCA
KRT6C	CD1C	KRT17	PCSK9	GSTA1	TTN	GSTA1	STAT3	PLCL1	MATN3	ARHGDIB	PLA2G5
SPP1	APOH	KRT6A	SFTPA1	FAM81B	ADM	EYA2	LIF	CCND2	SEPP1	ARAP2	PTGS1
PSCA	HSD17B6	KRT5	SFTPA2	C20orf85	SAMSN1	SIX1	LIFR	BCHE	COL3A1	PNMA2	CXCL5
PIP	DDC	KRT14	SLCO1A2	CAPS	THBS1	COL1A2	SPAG9	HHIP-AS1	FAM162B	CYFIP2	GNAI1
COL17A1	AGTR2	KRT6C	SLC26A9	PROM1	IL18R1	COL1A1	MYC	HHIP	CES1	IKZF2	MAP3K5
KRT5	ZBED2	THBS2	PLA2G1B	DYNLRB2	IL1RL1	COL3A1	PTPRM	GSTT1	GAS2L3	IKZF1	SOCS3
SNORD14E	MT1E	SPP1	CXCL2	SCGB1A1	RNASE2	AGBL2	PPFIBP1	PTHLH	PLK2	PRKCB	CD36
KLK12	HHIP	COL5A2	AGTR2	RSPH1	CHI3L2	CXCL12	OSM	CYFIP2	ARHGAP6	EPB41	HSD11B1
CXCL6	TNNC1	PLA2G7	CHRM3	CCDC113	SLCO4A1	CXCL10	TFPI	HERC5	THRB	GAP43	DPYSL4
COL3A1	ECEL1P2	PLA2G2A	DDC	MMP10	HNRNPU	CXCL6	CXCR2	ATP2A3	SYTL2	CXCR4	SEMA3A
OLFM4	F11	HTR2A	HDC	LRRC46	LOC100288	APLNR	CXCL3	BACH2	SNCAIP	DEF6	COL3A1
CYP24A1	DPP6	MAP1A	FOXA2	ZBBX	FIGF	ST6GALNA	IL8	SYT1	CLDN23	ZAP70	PCOLCE
PODNL1	C8B	KRT6B	HLA-DQA2	SCGB3A1	CTH	GALNT6	CCL20	DSP	MXRA5	SH2D1A	FGF7
KRT6B	BDNF	KRT15	SCTR	COL1A1	ZNF385B	IGF1	AGTR1	SCN2A	IL7	UBASH3A	THBS2
TMPRSS4	CLDN18	IGF1	ADRB2	HS6ST2	ORM2	IGFBP4	ADORA3	TMEM170	TEK	ADRBK2	ARHGAP6
CRLF1	SGCG	MMP1	CCR7	COMP	SERPINA3	IGFBP2	MAFF	EPB41	MASP1	TAL1	CARD16
AKR1B10	CACNA2D2	PAPPA	WNT3A	FAM216B	FMO5	CCL5	NFE2	CXorf57	GPNMB	SATB1	ANGPT1
LINC01127	PLA2G1B	MMP7	TNNC1	CFAP43	CCDC141	COL5A1	SOD2	KRT34	CD36	LEF1	TEK
LCN2	AGER	CYP19A1	TNNT1	S100A2	TIMP4	NME5	GPX3	SLC1A4	SNCA	ADA	LAMA2
PLEKHS1	CHIA	UGT1A6	APLN	DNAH12	PLA2G1B	DYDC2	TNFRSF11	FGF1	ADAMTS5	RAG1	LAMB1

TSPAN19	HMGCS2	CCNA2	CYP3A5	ASPN	CLEC4D	ALDH1A3	IRAK3	NRROS	RASGRP3	RAG2	C1R
CTHRC1	MYH2	CCNA1	FZD5	KRT5	OLAH	ALDH3A1	IRAK2	TGFA	CRISPLD2	SLA	MASP1
PROM1	SLCO1A2	VCAN	YTHDC1	SPATA18	SLC7A11	BGN	PLA2G1B	NAP1L3	LDB2	GATA3	CXCL12
S100A2	CNTN6	LTF	KHDRBS2	IGFBP2	SOCS3	UGT1A3	CSF3R	MFAP3L	RUNX2	CD93	PRKD1
MMP10	LNX2	CP	CCR3	CAPSL	ADORA3	DNM1	CYP3A5	FERMT1	TRPC6	PTPRCAP	EPAS1
HIST1H3D	C4BPA	UGT2B17	CHRM2	PLEKHS1	GPIHBP1	KIAA1377	ORM2	DSG2	SEMA3A	NKD2	BDKRB2
TIMP4	RS1	COL17A1	CXCL3	SPAG6	ANKRD12	UGT1A6	ORM1	PLS1	LRRN3	TGFA	ITGA1
PLA2G2A	ODAM	DST	CA4	COL15A1	PTPRB	CXCL11	JUN	CCNE2	SERPINF1	PECAM1	EDNRB
KRT17	CCL17	MMP3	CA2	KRT15	ST6GALNA	MS4A2	AGER	ENPP4	TMEM155	DSP	DCN
SIX4	CA4	CCL13	CYP3A7	ERICH3	NFKBIZ	FCER1A	S100A12	KIF21A	LRCH2	SEMA4D	WISP1
CHST6	LRRN4	CCL7	RASGRF1	TPPP3	TMEFF2	BDKRB2	MXD1	FAM221A	EYA4	SKP2	NQO1
LGALS7	SFTPA1	SPOCK1	DLC1	CCDC78	SDR16C5	KRT14	S100A8	SLC7A5	PTGS1	MYB	RUNX2
GPR87	RBP2	ACAN	BMP2	C9orf135	RTKN2	AKAP14	S100A9	MX2	CARD16	CCND2	FOS
KRT14	CPB2	HIST1H2BJ	SOSTDC1	CRIP1	HAL	MYCBP	SLC2A14	DHRS9	PLA2G5	FGF9	MAOA
COL10A1	SLC6A4	LOX	AMBP	CHST9	VNN2	ALOX15	SLC2A3	DPY19L2P2	PEAR1	PRKCQ	HNMT
PCSK1	GPR158	PSAT1	PARD6B	UBD	CPB2	SDC1	MGAM	BAALC	SPON2	ARHGAP9	IL13RA2
CP	FCER1A	SLC1A3	DMBT1	ABCA13	CXCR2	CXCL13	ARG1	SLCO4A1	TOX2	SLC7A3	HTR2B
UGT1A6	SFTPD	MAP1B	HLA-DQA1	TMEM190	SIGLEC10	CCL19	SLC6A14	MEF2C	PSG1	SLC1A4	TBXAS1
HSPA4L	LEPREL1	CCR2	SLC22A3	PSD3	EDNRB	EFNA5	HNRNPU	ICAM2	FAM155A	NOTCH1	FBLN1
HTR2A	CTNND2	DISC1	FOS	SERPINB5	HIF3A	EPHA3	FUS	TNFAIP8	TSLP	PTPN22	NR2F1
THBS2	WIF1	MMP13	NOS1	EPHA3	FAM76B	MAP1A	CBS	CELF2	RGS17	LMNB1	LPAR1
LRRC74B	LINC00312	SRD5A2	FZD8	PROM2	PKHD1L1	CYP4X1	CTH	FNBP1L	DLL1	RASGRP1	LAMA4
TNS4	EPB41L5	HSPA1A	WNT7A	CLCA2	RGL4	CTSD	MAOA	SLC16A9	F2RL2	DDX3Y	LDB2
P4HA3	PEBP4	WDR16	NFATC2	COL3A1	CLEC4E	ITGB8	CHRM3	KLHL13	HGF	STRBP	LMO4
ACAN	FAM189A2	S100P	IL13	CDHR3	CBS	TGFB1	AOX1	CHST11	MME	SLA2	DPT
CLCA2	SFTPA2	S100A2	WIF1	CTHRC1	CA4	MYH11	AASS	LINC01003	AREG	MAP4K1	ALDH1A3
CLEC4G	GPM6A	CHST6	COL4A3	CXCL6	SLC39A8	TPM4	SLC1A1	PARP8	SNAI2	HCLS1	LAMC1
RHOV	ODF3L1	B3GNT3	COL4A4	GPR87	MMP8	SLC6A1	SLC7A11	C4orf32	SLC38A4	HMHA1	SST
GALNT15	LAMP3	EYA1	IL6ST	C12orf75	FAM107A	GABBR2	IL6ST	MFAP5	F3	SLC16A10	IL7R
GSTA2	LPPR1	SIX1	CAV3	AKAP14	PEBP4	MFAP2	HAL	BCL2	PDGFD	SLC7A5	TSLP
FAM216B	IL13	TNFAIP6	CETP	WDR66	LOC10051	CFB	INMT	PECAM1	LAMB1	SKAP1	CHN1
CCNO	EFR3B	CENPF	FGA	IL13RA2	EGR1	C6	CA4	FERMT3	SEMA6D	PARD6B	ANGPT2
LGALS7B	AADAC	CENPA	CACNA2D2	PPAP2C	SAMD5	ITGB4	CA3	SPINT2	TMTC1	BCL2	F2RL2
MMP16	SLC5A9	DLG2	SFTPD	LRRC17	TBX5	CPE	SOCS3	RNF125	TMEM98	ITPR2	F2R
GSTA5	LRRC36	CENPE	COL6A6	MS4A8	NFE2	ROBO2	ETS2	ST6GAL1	ZNF608	TLE1	GRK5
COL1A1	LINC00968	MFAP5	EDNRB	SPP1	FMNL2	ABCC3	BTG1	SCD5	FGF7	GNAS	ROBO1
SPOCK1	TINCR	FBN2	MALL	GABBR2	FAM65B	FABP6	DUSP1	EAF2	CPED1	PTHLH	COL5A2
POSTN	EMP2	UCHL1	NOS2	C7orf57	MT1M	KRT17	CDKN1C	USP1	PCDH18	CDO1	EGR1
ZBBX	CDH16	MMP2	PTGS2	C1orf194	EPB41L5	KRT6A	NR4A2	BTBD11	TYRP1	CCNE2	PDE4B
LTF	ANGPTL7	LCN2	TNNT2	EFHB	CCDC85A	HTRA1	HIST1H2B	TLE1	KRT7	CDC25A	AK5
SCG5	SLC26A9	TIMP4	SH3GL3	WDR78	HSPA1B	HS6ST2	BCL10	KCNN4	LOC100128	SLC6A15	AOX1
MMP13	XCL1	RHOV	HAS1	PDLIM4	APOBEC3A	HS3ST1	INSIG1	NMI	METTL7A	ARHGAP26	COL4A1
F2RL2	HAGLR	ARHGAP11	HAS3	VWA3B	NLRP3	CYP2S1	HMGCR	FAM171A1	SGIP1	LCP2	COL4A2
FCRL5	SHISA2	BIRC5	ADRB1	KLHL13	STRAP	PKP2	ARHGAP6	OCIAD2	MYOCD	DTL	PTGDS
RARRES1	ASPG	CRYAB	BMPER	CFAP53	SLCO4C1	IGFBP7	RHOH	ITPR2	TFPI	WDR76	S1PR3
MAP3K19	IGFALS	CYP2F1	NR0B2	TMEM232	S100A8	CLCA2	PTPRG	EML6	CYB5R2	CDT1	TGFB2
HIST1H2AN	CCK	SERPINB13	PLA2G12B	DNAI2	CD69	TRBC1	B2M	ZNRF1	AMPH	TFDP2	ASL
PTPRT	CYP3A4	CTSB	RAMP2	LPPR4	HHIP	HLA-DQA1	ND6	ZNF529	PTN	PITX2	GPX3
APOD	FAM105A	COL7A1	SHANK2	MUC16	PTPRG	THBS2	CYCS	KBTBD8	SOCS3	HERC5	NID1
DNAH3	C2orf54	FBLN2	BAIAP2	WDR16	GPR97	MYB	TLR4	KCNC4	FOXF2	PIK3R5	CDKN2A
MS4A6E	KRT72	HMMR	IL6R	IQCA1	FCAR	CD4	PTGS2	STK17B	IL1R1	MEF2C	RASGRF2
CFAP53	HDC	TPX2	DAO	MNS1	CCL20	KRT15	HMOX1	ING5	CDKN1C	ELMO1	ARHGAP28
CENPA	TNNT1	TIMP1	SLC1A1	DST	BCL10	COL6A1	SF1	CDO1	HEY1	DOCK2	CASP1
DYDC2	WNT3A	BUB1	SLC1A2	FNDC1	ND6	FOXA1	HMGCS1	GALNT7	ELMOD1	ITGB2	EGFR
C20orf85	NR0B2	FOXM1	CXCL11	MYH11	EMP1	SFRP2	FOXO1	CDK6	PSG5	GCH1	ADARB1
KIF26B	PARD6B	NEK2	PVRL3	SPATA17	ORM1	FZD3	ATF3	TM6SF1	MAP3K5	GCHFR	ROBO2
ENKUR	PRTG	SRGAP3	CADM1	CCDC170	ACADL	LDOC1	ACSL1	GNAS	NDNF	ADK	ALDH1A1
GREM1	RASGRF1	COL5A1	PENK	COL1A2	INSIG1	PTK2	ACADL	LRRC8B	HNMT	NMI	GAB1
FGF7	ADRB2	CDC25C	TIMP3	CD24	MYLIP	CCL21	OLAH	GDAP1	GNAI1	AGTR1	HGF
TTC29	SLC46A2	NUF2	DHCR24	TEKT1	CAMK2N1	NTRK2	ACAT2	HMGCS1	ANO4	INPP5D	CACNA2D3
MUC16	RGS9BP	SLC40A1	TGFB2	KCNJ16	FPR2	DOK5	TIMP3	PDE7A	SLC35F3	MCM10	RASGRP3
EFNB3	COL4A3	CYP2A6	TPM3	CAPS2	CLDN18	PCSK2	P2RY1	FAM149A	DLX2	DEPDC1B	PTGER2
PAPPA	TMED6	GOLM1	CYP4B1	LOC10192	XYLT1	SCG5	IL18R1	TRIB3	TMEM220	PLCH1	BTG1
SPAG17	GRM8	CEP152	ADAMTS1	CFAP70	PTPN12	TF	PTX3	ANKRD44	DCHS1	SCD5	THRB

MUCL1	MYRF	AGT	VEGFA	SLC44A4	CD177	FGF14	FGF2	ADK	SRPX2	GAS7	AHR
KIF20A	SYNDIG1L	BUB1B	SULT1A2	MUC4	BOD1L1	FGFR3	GRIA1	ZCCHC2	RBMS3	APBB1IP	CD44
PIH1D3	ADAMTS8	DEPDC1B	ARAP2	ITLN1	RC3H1	SFRP4	ADM	ADCK2	FLRT3	HMGB2	SRGN
CRYAB	LOC10013	HIST1H2AD	PLA2G3	C11orf88	LOC10028	SIX4	ADRB2	DENND1B	TOR4A	TCF7	CHRM2
SCG2	SEMA3E	IGFBP2	PON3	TP63	FUS	DDR1	TLR2	GHR	LINC01140	CDC6	PRKAR2B
DPEP1	LINC00165	COL6A3	FOLR1	CASC1	BTNL8	PTPN13	FPR1	TIMP3	NCKAP5	PLCL1	MATN2
DLGAP5	PCSK9	CXCL5	FOLR3	GREM1	CCK	PDLIM4	ANXA1	TPK1	PSG9	GIT1	MATN3
ARMC3	EFCC1	TOP2A	ADCY8	SERPINB3	ARHGAP6	ENAH	FOSB	ACAT2	KCNE4	ITPKB	KITLG
CCDC37	FAM71A	CDT1	CPAMD8	KIAA1377	FIBIN	EPHX2	SERPINA3	CYP4V2	BNIP3	GRIA3	SNAI2
TM4SF19	FHDC1	ITGA11	SOCS2	ST6GALNA	GALNT15	KCNMA1	HSP90B1	CCDC109B	LINC00619	POLE2	ACTA2
FAM92B	FAM167A	MAD2L1	IL20RA	PTGFRN	INO80D	ROBO1	FPR2	CDON	UNC5C	PRIM1	RGS5
IL13RA2	HOXA6	MMP14	TRIM63	C1orf192	SCN7A	BSG	AKAP12	BRI3BP	LAMA2	CD1D	MITF
CCL7	MAPK4	BCL2L11	SLC14A1	SCG5	DUSP1	KRT19	PRELP	PPM1K	LOC40004	KAT2B	TYRP1
GSTA1	PLA2G4F	NGFR	SLC5A1	ITGBL1	SELL	ACE	NID1	ZPLD1	GNG11	RHOF	PLAU
DQX1	ARC	ABCC5	CEBPB	MMP12	NAMPT	COL6A2	PTPRB	AGTPBP1	HTRA3	PAWR	TNFRSF11B
CEMIP	PPP4R4	PDE1A	PRKCQ	TRIM29	PHACTR1	FN1	TEK	ZSWIM7	POSTN	WT1	AKR1C3
TDO2	HTR3C	RIBC2	SDC4	SLC28A3	FPR1	EYA4	ADRB1	CXADR	ALDH1A3	TTF2	ARNT2
ATP12A	ZNF385B	MYBL2	VPRBP	COL14A1	FKBP5	SPAG16	TNNC1	RORB	PTGR1	PTPN3	DMD
CILP	NAPSA	DEPDC1	CUL3	DNAI1	WWC2	SPAG6	TTN	MAP3K9	WISP1	ALDH5A1	FGF2
MMP3	GPIHBP1	GGT5	HOMER1	CATSPERD	LIFR	ITGA9	AOC3	PTPRR	EPAS1	OXCT1	ST6GALNA
MGC50722	SLC14A1	KIF2C	GRIA1	PIFO	CXCL3	COMP	S1PR1	ATF3	GALNT15	SIT1	KRT7
ADAMTS16	FGFBP2	C6orf165	SLC5A9	PRSS12	STARD13-A	SCGB1A1	MAF	DHCR24	ROR2	PPP1R14A	SEPP1
MCHR1	CADM1	TP73	GBA3	CCDC74B	MAOA	FGG	APOA2	ENDOD1	PXDN	E2F2	HLA-DPB1
KIF11	EMP1	CDCA8	GP1BA	ZMAT3	EIF1	SLC44A4	LDLR	TAP2	ANGPT1	ICAM2	MYLK
CCL18	KIAA1683	CDK1	F11	SCGB2A1	FAM150B	SLC44A5	ZBTB16	ZXDC	KCNJ15	CBFA2T3	COL1A2
TOP2A	NAPSB	KIF11	KCNJ5	SIX1	FLT1	FGFR2	MAP2K6	FZD5	AKR1C3	FNBP1L	C1S
NPTX2	OLFML2A	RAD54B	FCER1A	TSPAN1	MCL1	KRT8	SLCO2A1	PSAT1	RNF182	CDK6	ALDH7A1
C6	CLIC5	CHEK1	MS4A2	C9orf117	FOS	AKR7A2	SLCO4A1	RHOF	WDR35	CPE	ABCC4
C11orf88	GRIA1	SLC7A11	CYP2C18	CHIT1	RNF182	GGT6	ARHGAP29	GPC4	ITGA1	PMCH	LRCH2
ADGB	DNASE1L3	COL1A2	INADL	ITGB8	CXCL8	CARM1	PTPN12	MARS	RGCC	ESM1	MPDZ
MMP7	SDR16C5	TEKT4	KCNA4	SCARA3	CD274	MMP2	NEDD9	SNHG17	DHRS3	NKX2-5	HSPA2
SOX2	SHANK2	CENPM	ACTN2	SPAG17	AGTR1	LCN2	EPAS1	TSEN2	HTR2B	DIAPH1	MAPK10
IGFL2	CHRM3	CCL16	PPP1R1B	PCDH7	RXFP1	COL17A1	FLT1	SNHG15	AOX1	PAX6	CREB5
CCDC17	KCTD16	HGF	AMOTL1	ARMC3	PGC	CYP2J2	SULT1B1	DOCK9	DCN	CDC7	PPARGC1A
STEAP1	LINC00472	PLD1	PLK3	MORN5	HSD17B6	WNT4	PAPSS2	TSHZ1	FOXF1	NCAM1	SERPING1
KCNQ1OT1	ACADL	HS3ST2	KRT72	WDR38	B2M	SORD	HNRNPD	LONRF1	CLEC2B	ST8SIA4	COL6A2
CXCL12	ZBTB24	HS3ST3A1	KRT18	CP	LINC00622	TDO2	SLC26A2	DDX50	ST6GALNA	RBBP4	PTK2
SPRR1B	MEGF11	ALOX15	ARHGEF4	ODF3B	CASS4	CALM3	PIM1	PSTPIP2	FAM13A	BCL11B	AMPH
WDR16	PDZD2	PBK	MYC	CDH3	ETS2	INO80B	RPS6KA2	BAG1	RCAN2	ETS1	MMP3
CCNA1	MS4A15	DIO2	RHOBTB2	TTC29	MXD1	NAT1	FYN	EPB41L4B	NID1	SOX3	CCL2
SLC16A10	YTHDC1	DIO1	SPOCK2	FGF14	EMR2	TRIM29	DLC1	GATSL3	CSRP2	ARHGAP4	IL6ST
C9orf117	HLA-DQA2	B4GALNT1	TES	C2orf40	LMNB1	TSGA10	TOP1	SQLE	FLJ35700	OPRM1	JAK1
MUC4	HLA-DQA1	MAT1A	SLC6A14	AGBL2	CR1	CD74	AKT3	SLC3A2	CASP1	CCR5	GUCY1B3
SLC51A	C16orf89	PLOD2	IGF2BP1	IGFBP4	AOX1	SLC28A3	MAP3K8	ZFP3	RAB31	DOCK8	LAMA1
C2orf73	SOSTDC1	DIO3	C1orf116	CNTN3	CYP3A5	TYMP	HHEX	CXXC5	FMO3	RHOJ	CAV1
CCDC170	SPRYD7	FBN1	CDH1	ALDH1A3	TFPI	ACTA2	ARHGAP26	GK5	B3GALTL	DNA2	TWIST1
KRT23	CALB2	C6	RGS9BP	SLITRK6	SOX7	FNBP1	CD44	RBBP4	LOC100506	MCM6	PXDN
NGFR	CLIC3	C7	PDE6A	COL10A1	LYVE1	GPX7	BCL6	BMP2K	YPEL4	FN1	TRPC4
ABCA13	EIF5	SHCBP1	CYP4Z1	ZMYND10	CCBE1	TCF7L2	ARHGEF7	CCDC181	PROCR	SLC3A2	TRPC6
SCRG1	GDF10	PLK1	MIP	LCN2	GPM6B	EVL	PDE4B	ETV6	LPAR1	ITPR1	TBL1X
SPAG6	DHRS2	KIF20A	VIPR1	CXCL13	BTG1	VCAM1	RAPGEF4	ANAPC7	SLC16A4	MAGI3	KIT
DRC1	ABCA3	SRPX2	PIP5K1B	SLFN13	MAFF	LGALS3BP	TXNRD1	PCM1	CD248	E2F5	PYGL
FAM81A	SLC39A8	MKI67	OCLN	EPHX1	IRAK3	ERBB2	TXN	GTPBP3	CTSC	PPM1B	CALD1
MS4A8	PGC	DDX3Y	NR4A1	CYP2F1	IER2	ADRA2A	EDNRB	JADE1	CDKN2A	LMO2	RIPK2
C1orf194	NCKAP5	MFAP2	NKX2-1	CXCL10	KLF2	ROGDI	GNAQ	ZNF83	NQO1	TUBA4A	PRKAA2

IPF stage 1 vs normal											
GSE10667 - stable				GSE24206 - early				GSE44723 - slow			
Limma		NetWalker		Limma		NetWalker		Limma		NetWalker	
up	down	up	down	up	down	up	down	up	down	up	down
COL17A1	ITLN2	KRT6B	IL6	MMP7	S100A12	MMP7	IL6	DSP	SLITRK6	MGP	CDCA8
MMP1	CHIAP2	KRT15	SH3GL2	SERPIND1	IL1R2	SPP1	ZBTB16	MFAP5	KIF20A	BMP2	BIRC5
COMP	SLC6A4	IVL	IL6ST	SFRP2	MT1M	MMP1	FOS	IL24	IQGAP2	CYP1B1	KIF23
KRT6A	RTKN2	KRT1	SOSTDC1	COL1A1	IL6	PTPRZ1	CEBPD	DSG2	ARHGAP28	GSTT1	KIF2C

IGFL2	SERTM1	IGHG1	BMP2	COL3A1	ARG1	MDK	STAT3	MFAP3L	TMSB15A	PLA2G4A	SGOL2
UGT1A6	HTR3C	IGKC	EDNRB	CXCL14	XIST	CXCL13	IL6R	GSTT1	HMMR	PTGS2	NDC80
MMP10	AGBL1	KRT14	CAV1	LPPR4	MGAM	CCL19	TSC22D3	HECW2	TOP2A	SPP1	SPC24
SPP1	CA4	KRT5	EDN1	NTM	PTX3	MS4A2	LIF	EPHA5	CDKN3	THBS1	KIF18A
KRT5	KLRG2	EYA1	IL6R	RPS4Y1	SLCO4A1	FCER1A	LIFR	ICAM1	DEPDC1	FN1	NUF2
BPIFB1	SOSTDC1	SIX1	CER1	CXCL13	IL18RAP	CCND1	IL1R2	FNDC1	FAM83D	APBB1IP	CENPH
IL13RA2	NECAB1	UGT1A6	WNT3A	UBD	PROK2	XPO1	IL1RAP	CPE	KIF4A	TRIB3	SPC25
GPR87	SLCO1A2	CYP2F1	COL6A6	ASPN	THBS1	KRT5	CSF3R	SPP1	MASP1	IGFBP3	CENPF
PCSK1	BTNL9	TUBB2B	COL4A3	COL15A1	DEFA1B	TP63	PLA2G1B	MGP	CCNB2	CADM1	CENPE
RPS4Y1	SLC5A9	TUBB3	ALOX12	FCER1A	CLEC4E	COL1A2	SOCS3	SERPINB2	BIRC5	EPB41L3	CENPA
GREM1	CRTAC1	CHST6	CYP3A7	CTHRC1	CXCL8	COL1A1	IL6ST	APBB1IP	MAP2	COL13A1	RACGAP1
TMPRSS4	MYRF	B3GNT3	CYP3A4	DDX3Y	APOLD1	COL3A1	MAFF	IL33	CDK1	SLC6A15	CDK1
RPS4Y2	GPR158	IGHA1	CYP3A5	CHIT1	LOC100288	SOS1	NFE2	MN1	METTL7A	SLC1A4	TOP2A
MMP13	DPP6	GSTA2	PLA2G1B	HS6ST2	DUSP1	APLNR	SAP30	PTPRB	GPR126	CDO1	KIF11
MMP7	GRIA1	GSTA1	PLA2G12B	EPHA3	FPR1	PPBP	MXD1	GATA6	PRC1	FGF1	LMNB1
KRT14	CYP3A7	GSTA5	MME	USP9Y	SERPINE1	CXCL10	ORM1	OXTR	CENPE	SYT1	RRM2
SERPINB3	VIPR1	GSTA3	WNT7A	ITLN1	ADM	CXCL5	SERPINE1	PTHLH	HOPX	PAPPA2	PTTG1
CRLF1	F11	KRT6C	SH3GL3	PROM2	NAMPT	CXCL12	ORM2	MEDAG	CCNA2	CPE	PBK
PLEKHS1	IL13	KRT17	HLA-DQA1	CDCA7	FKBP5	IGF1	KLF6	KRT34	CCNB1	PMCH	CEP55
KRT17	ECEL1P2	KRT6A	AGTR2	COL14A1	FPR2	IGFBP2	KLF4	IL6	SYTL5	PLAT	CDKN3
SCRG1	CTNND2	ALDH3A1	ADCY8	ITGBL1	SOD2	TRBC1	ACSL1	SLC39A8	SCN4B	IL6	CCNA2
BPIFA1	LINC00968	SRD5A2	APLN	CRIP1	CSRNP1	HLA-DOB	ACADL	CCND2	MIR155HG	PTHLH	CCNB1
SCG5	RS1	BAAT	CHRM3	ROBO2	NR4A2	ROBO1	OLAH	GCH1	KIF14	SFRP4	CDC25C
PLA2G2A	AGER	SLC27A2	VIPR1	FNDC1	VNN2	UGT1A3	PTPN11	NTN4	MYOCD	FZD8	CCNB2
SFRP2	CYP3A5	PLA2G2A	ADRB2	S100A2	APOBEC3A	CYP2F1	SOD2	MYO1D	AKR1C3	CBS	SPAG5
KRT6C	HSD17B6	NGEF	ADRB1	KRT17	CXCR2	CXCL9	GPX3	SULF1	IL7	BMPER	MAD2L1
KLK12	CCK	RHOV	NOS2	CX3CR1	FOSB	HLA-DOA	S100A8	CNIH3	NEK2	BMP6	PRC1
SERPINB4	ESM1	IGF1	HLA-DRB5	COL1A2	SERPINA3	GSTA1	S100A9	GRIK2	CDCA3	TGM2	KIF4A
COL1A1	HMGCS2	MMP1	TNNC1	MGP	MXD1	COL5A1	CXCR2	TLR4	SLC38A4	PDGFA	SLC38A4
LGALS7B	FGFBP2	UGT2B17	TNNT1	COL10A1	IL18R1	DDX3Y	CXCL2	SCG5	CENPF	ARHGAP26	KYNU
SAA4	PLA2G1B	COL1A1	FZD5	KDM5D	CLEC4D	MFAP2	CXCL3	PTPRR	CLDN23	FGD4	CASC5
MMP11	ADRB1	THBS2	CA4	COMP	MAFF	SDC1	IL8	STK26	IFIT1	FBLN2	AURKA
ADAMTS16	CYP3A4	SPP1	CA2	THY1	ORM2	LEF1	MYC	PNMAL1	DEPDC1B	ARAP2	UBE2C
LGALS7	FENDRR	COL3A1	NR3C1	LRRC17	PADI4	KRT14	CXCR4	STC2	NUSAP1	LIPG	TACC3
TMEM229A	SLC14A1	CYP2A6	BMPER	GPR87	TIMP4	ROBO2	CCL20	BMP2	SPAG5	PNPLA3	HMMR
HS6ST2	BDNF	TP63	CXCL3	CBLN4	SLC7A11	TOP2A	HNRNPU	ARL4C	ST6GALNA	PECAM1	TPX2
MMP3	C8B	THBS4	CXCL2	APLNR	PGC	ABCC5	HBEGF	AADAC	TJP2	DSP	PRKAR2B
AKR1B10	TINCR	PNOC	ADAMTS1	C11orf80	CEBPD	PDE1A	CBS	CCL20	ACTC1	PTGIS	PLK4
VTCN1	PRX	CXCL6	VEGFA	CXCL5	IL1RL1	RGS5	CTH	EMB	LOC645722	FNBP1L	NEK2
CILP	WNT3A	IGFBP5	VIP	MUC5B	HAL	GNAI1	SLC6A14	ANKRD1	PTTG1	PTGDS	FOXM1
CHRDL2	KCTD16	IGLV1-44	RGS9BP	PCDH7	CXCL2	EVL	SLC2A14	BAALC	KIF15	PTPRR	ZWINT
OGDHL	GPM6A	HTR2A	RGS9	MS4A2	SIK1	CCL5	SLC2A3	DPP4	CENPW	CDH2	THRB
CDH3	CLDN18	MAP1A	NOS1	SLITRK6	SLC19A2	MSH2	MGAM	PPAPDC1A	FLRT3	MGLL	TRIP13
SLN	LRRC36	CXCR5	CHRM2	VASH1	ZBTB16	IGFBP4	NFIL3	PCDH10	HJURP	IGF1	CLEC3B
CXCL14	NCKAP5	GNAO1	SPOCK2	UGT1A3	MYC	GUCY1A3	CREM	KIF21A	FOXM1	GREM1	HGF
SIX4	FIGF	MMP7	PLXNB3	NREP	ADAMTS1	COL6A6	FOXO1	CHST15	MATN3	CD58	TTF2
KRT15	BTNL3	ACAN	EDN3	SPATA18	FAM107A	CPE	USP53	ZPLD1	SGOL2	HERPUD1	PDE3B
CXCL13	KIR2DS4	SRGAP3	CTNND2	HOXC6	RGS1	GUCY1A2	ARG1	ITGA11	PLK4	SLC7A5	CEP70
PODNL1	SEMA3E	PCSK2	PDZD2	SLC28A3	MT1X	CCL13	BCL6	KRT19	OIP5	JUN	DHFR
S100A2	HHIP	SCG5	DAO	CD24	CCL20	KRT17	MAOA	KCNK6	TNFRSF19	CTH	TYMS
TNS4	TMEM100	DST	SLC6A14	GABBR2	FMO5	KRT6A	CEBPB	FUCA1	EYA4	SPARC	SHCBP1
GSTA2	DEFA4	LTF	SFTPA1	CXCL10	NR4A3	NUP210	HAL	CSGALNAC	MND1	XDH	TK1
LCN2	ASPG	CP	SFTPA2	CDH3	AFF3	MOXD1	AASS	SYT1	KIF18A	GPX7	MELK
COL10A1	NCR1	PSAT1	FGA	TP63	ERRFI1	SLC18A2	SLC7A11	KCNJ2	IQGAP3	CCND2	TMPO
PROM1	SGCG	BBOX1	KHDRBS2	ECM2	TTN	PRKACB	ALOX15B	FZD8	KIF18B	CDK6	KIF15
SOWAHA	LOC100131	EPHA3	CADM1	ITGB8	GADD45B	UGT1A6	GFPT2	CXCL8	CDCA7	BCAT1	SELENBP1
SERPINA5	TNNT1	CCL7	MPP3	KRT15	RNASE2	ITGA2	ARG2	RNF128	KIF2C	INHBA	BUB1
CTHRC1	DAO	IGKV1-5	CD274	IL13RA2	SPIDR	ZBTB33	PFKFB3	TENM3	RGS17	IGFBP7	CENPM
CXCL6	NXF3	FBLN2	EPAS1	MXRA5	MT2A	ENAH	SOCS2	SLC20A1	LMNB1	COL5A1	CKS1B
BAAT	MT1E	COL17A1	NFKB2	CXCL12	NLRP3	LCK	IRS2	CD55	CDC25C	HDAC9	HNMT
DIO2	EFR3B	ITGA11	CDH10	MS4A1	CYP4F3	TF	BCL3	CCDC85A	CDCA8	SPEN	MAOA
GJB2	DEFA3	GGT5	PTN	CCL18	RGS2	CENPK	PIK3R3	DNAJB9	SOX6	CER1	MKI67
P4HA3	MYZAP	SAA2	SLCO1A2	FAM13C	PHACTR2	SMAD1	THBS1	FN1	SYTL2	COL5A3	MATN2
GSTA5	APLN	LAMA1	SLC26A9	ANO1	SAMSN1	CXCL11	TNFAIP6	IRX3	TSLP	HTR2A	MATN3

KIF26B	ANKRD1	CCL13	TES	LRRN1	FAM65B	IGKV4-1	FLT1	PLA2G4A	KIF23	TRIO	BARD1
SPRR1A	FAM167A	RIBC2	RAMP2	FRZB	CHI3L2	TGFB2	SELL	ANKH	PTGER2	MORF4L2	PTN
MMP16	ARC	MMP13	GRIA1	RGS13	AREG	LTBP3	SELE	GRIA3	SESN3	LAMA1	FANCD2
HCAR1	MS4A15	PROC	HOMER1	DCLK1	SDR16C5	MASP1	CH25H	NTM	PSRC1	HIST2H2BE	RUNX2
MSMB	TNNC1	SERPINA5	COL4A4	TXLNGY	C11orf96	KRT15	LDLR	PTGFRN	DHFR	CSTA	ERCC6L
COL3A1	ANGPTL7	SAA1	SPHK1	TRBC1	MT1H	THBS2	ETS2	CYP1B1	PLA2G5	PTGES	NCAPD2
GSTA1	STC1	EFNB3	PTGS2	SLC4A11	NFKBIZ	THY1	JAK1	LRRN4CL	ARHGAP11	CD55	CENPN
TTR	KIR2DS2	RHBDL2	PDGFB	ST6GALNA	CD69	ZNF521	IER3	SLC6A15	MAB21L1	C3	MLF1IP
POSTN	GALNT13	MMP3	C8A	TPPP3	SLC2A14	PDE5A	MCL1	FMOD	SPC24	ACOT4	CENPI
PSCA	AGTR2	SPOCK1	C8B	FFAR4	SOCS3	ST6GALNA	PMAIP1	KCNC4	FANCD2	COMP	BLM
TUBB3	RBP2	GRIN2A	ZBTB16	VSIG1	S100A9	GALNT6	OSM	HDAC9	COL3A1	RPS4Y1	ITGB3BP
PCP4	TMSB15A	CXCL5	FZD8	CIRBP	HIF3A	DEPDC7	SLC7A2	THBS1	CKAP2L	SSR1	MCM10
ATP12A	CNTN6	DLG2	SLIT2	ZBED8	KLF9	PDE7B	FASN	ELFN2	ASPM	LRP8	COL3A1
SAA1	KIR3DL2	COL7A1	HMGCS2	GPX8	BRE-AS1	EPHX1	ATF3	SHROOM2	TRIM59	RELN	PCOLCE
PIP	COL4A3	DES	HMGCS1	TDO2	FAM150B	MUC16	PPP1R15A	GPC4	GBP2	CCL20	SNAP25
CP	SH3GL2	S100B	FUT1	PTGFRN	MT1G	MSLN	TFPI	CDO1	PTN	SSTR1	SNCAIP
SOX2	LNX2	MFAP5	AK5	NAPEPLD	PIGA	IGFBP7	PTGS2	ATF3	CLEC3B	CSGALNAC	NDE1
CLCA2	ZBED2	FBN1	SLC4A1	SCN4B	MT1E	PTN	IRAK3	TMEM171	PTPLAD2	CHST11	KIF20A
RAB3C	GRM8	BDKRB2	CEBPD	ZNF711	MT1HL1	HGF	IL1B	MXRA7	TACC3	GRIK2	AOX1
CYP24A1	FLJ34503	CDH2	TFAP2A	C12orf75	NFIL3	VCAM1	F5	CFI	CENPA	PTPRB	CXCL12
CDH2	RGS9BP	BOC	SLC6A4	SLAMF7	SELL	ITGA9	SERPINA3	KRTAP1-5	GPR85	F11R	C5
IGLL5	APOH	FBN2	STAT3	SCG5	GPR97	PTPN13	TOP1	TRIB3	ANO4	NCOA3	VAMP8
PIH1D3	FAM105A	TTR	ACE	PRSS12	EMP1	PDLIM4	MMP9	HERPUD1	NEIL3	FYN	PTCH1
PCDH7	CCDC85A	PHYHIP	GP1BA	HTR2B	ATP13A4-A	NDC80	MT2A	HEPH	ANO3	SQSTM1	ACTC1
PTPRT	CLIC5	TF	F11	COL8A2	MT1F	MDH1	AOX1	FGD4	PHF19	GRIA3	SYNE2
EFNB3	JPH1	AGT	ST6GALNA	IL33	ACSL1	MS4A1	JUNB	DACT1	MAOA	GPC4	NES
HTR2A	KRT72	CYP2A7	S1PR1	SULF1	MMP8	RAB30	SLC16A10	HTR2A	CEP70	FSTL3	LBR
ADAMTS14	S100A3	COL1A2	CSF3R	KIAA1377	SLC2A3	HLA-DQA2	NFKBIZ	IGFBP3	PDE3B	RPL27A	DCN
TUBB2B	KLRF1	IGFBP2	CEBPB	ADAM12	FCAR	HLA-DQB1	GRB10	COL13A1	MAD2L1	IGF1R	COL14A1
PSAT1	ODAM	COL6A3	SOCS2	ZNF521	ZFP36	HTRA1	NFKBIA	EPHB1	ZNF608	NEFL	MCM8
CHST6	LEPREL1	ADCY2	LIFR	FRAS1	SCN7A	ASPM	NFKB2	DUSP6	EPR1	DPP4	RFC3
CHST9	IL6	PLXNA4	DLL4	XPO1	RASD1	PENK	OSMR	AFAP1	GPR137C	FAP	DIAPH3
FAM83D	EDN1	DPYSL4	NOTCH4	BCL11A	LDLR	COL4A5	AGTR1	CALB2	CASC5	SLC3A2	KPNA2
TIMP4	EPB41L5	GPT2	USHBP1	EGFEM1P	CD163	CIITA	BCL10	SLC1A4	KIAA1524	NOX4	MAP2
CCNO	C21orf90	MMP2	FAM107A	ZMAT3	CSF3R	NTS	CYP4F3	EGR3	GAS2L3	TLR4	KNTC1
FAM150A	SH2D1B	LCN2	SLC9A3R2	CCND1	PER1	MYB	LTB4R	CADM1	NCAPG2	ICAM1	ZWILCH
IGHG1	CACNA2D2	TIMP4	GJB1	FAM162B	MFAP5	GPX7	CYP3A5	SYNDIG1	RAD54B	BMPR1B	STMN1
FAT2	DAPK2	CCR2	SPHKAP	TLR7	HSPA1B	PTPRO	IL18R1	FAM171A1	F2RL2	VDR	CHEK1
KRT6B	EMP2	FABP6	TAT	DIO2	ORM1	RASGRP1	FOSB	PLAT	TRPC6	ATF3	CENPK
SAA2	STXBP6	CFB	RAPGEF4	LINC00537	CCDC71L	CCR5	FOSL1	FGF1	PIR	JUND	ADSSL1
IGF1	CAMP	C6	PFKFB3	LINC01094	TACC2	MAD2L1	TLR4	MFGE8	DLEU2	HES1	FBXO5
C6	RXFP1	KRT81	FBP2	ACE	CMTM2	UBD	TLR2	CPEB4	CDKN2C	SLC38A1	CCNF
FAM92B	HPGD	ALDH1A3	RAMP3	VSNL1	RGL4	KLHL13	HP	SOD2	HGF	CHST7	UBE2T
ISM1	NMUR1	FLNC	DNM3	IGDCC4	SOCS2	FGFR3	CD163	DDX17	LOC10050	BST1	SST
CPNE4	EDNRB	CXCL13	CAV2	C15orf48	PELI1	KIAA1377	CXCR1	CBLB	TTF2	NMNAT2	DEPDC1
SPOCK1	LTK	CCL19	CACNG4	MIR99AHG	LOC10050	ATF7IP	CXCL1	CCPG1	MMD	EPRS	RHOU
B3GNT3	HECW2	DIO2	PRKCE	GZMA	S100A8	ZNF518A	IRAK2	TSHZ2	CKAP2	SSR3	DEPDC1B
MRAP	EFCC1	TPO	MYH2	KLHL13	ZNF385B	TNFSF13	PELI1	TGM2	FAM13A	IGFBP5	ARHGAP28
ERICH3	FCN3	ADH4	RGS16	MMP16	SLC6A14	CD4	ETS1	C8orf34	HR	MEF2A	ARHGAP11
CCNA1	SLC39A8	GSTT1	GNAT1	EIF1AY	CXCR4	TCF7L2	TNFRSF11	KCND2	ANGPT1	BDNF	DUT
DNAH3	KHDRBS2	CYP19A1	SLC5A9	PIEZO2	CRISPLD2	ZWINT	CYCS	CBS	SELENBP1	CXCL1	MYLK
ESR2	VEGFA	VCAN	SLC2A3	NBPF1	SIGLEC10	DNM1	BMX	CYP4V2	PARPBP	CD36	TGFBR1
HES2	IL1A	CYP2J2	GBA3	CNTN3	PDK4	IGHM	FOSL2	INHBA	TMPO	EIF1AY	MLLT3
TDO2	CD160	PAPPA	KLF6	FMO3	EGR3	IGJ	HIST1H2BC	SLC22A15	SSX2IP	ATXN1	TJP2
RARRES1	MATN3	FHL2	TIMP3	MARCO	GFPT2	TSPAN1	MAP2K6	NUAK1	HNMT	CXCL2	KBTBD7
EFCAB10	CLIC3	AGBL2	KCNG3	HLA-DOA	CCDC141	CTSD	HMGCS1	ASB1	LINC01140	SRD5A3	AMPH
IGHA1	DHRS2	PLN	KCNC1	PRSS2	IRS2	PLN	JUN	LOC15357	BORA	MARS	SOX6
SYT8	LGALS2	SLN	SLC1A1	MUC16	CTH	CTSK	HSPA1A	PTPN22	ZNF273	CYCS	GGH
IGLV6-57	PPP4R4	FBLN1	HHIP	KIAA1211	HNRNPU	HLA-DPB1	DNAJB1	ADAMTS12	LINC00960	RAC2	PENK
SIX1	CPB2	CXCL12	SHH	ANKRD36B	FFAR2	PLCB4	CDKN1A	FAM167A	KNSTRN	TGFB3	PRKACB
MUCL1	OLFML2A	ABCC3	FLT1	BCHE	LOC10050	HTR2B	GADD45B	TNFSF9	FOXF2	FMOD	RFC5
ERICH5	APOBEC3A	PLG	TEK	NAP1L3	STC2	CCNB1	CDA	LOX	CKS1B	NTS	SMC4
TSPAN19	ANKRD20A	MAGED4B	PTPRB	PLXDC1	MAOA	SPNS1	FUS	SAMD3	PSMB9	ABL2	NUP107
C9orf135	PEBP4	COL5A2	RASGRF1	RBM43	TNFAIP3	COL7A1	DDX3X	MAFF	MKI67	HSD11B1	MET

F2RL2	GALNT18	SLC1A3	SLC2A14	LOC100505	ATF3	FGF14	ACSL4	ARHGAP26	PLXNC1	ATF5	CEBPD
FNDC1	ANKRD29	ALOX15	SSTR1	TRIL	BTNL8	HS6ST2	PDE4B	FKBP11	TP53TG1	IGFBP1	MAP3K5
EYA1	AFF3	UCHL1	IL8	PSD3	OLAH	HS3ST1	PDE4D	CPPED1	PTTG3P	SLC7A7	RAB27A
ACAN	PCDH12	CBLC	ARAP2	GXYLT2	FLT1	COL5A2	ADM	ALDH1L2	GAS6-AS1	KCNJ2	SYTL5
ANXA13	GPIHBP1	IL13RA2	FGG	CAPS2	CBS	PLA2G4A	ADRB1	PLXNA2	KCNE4	DLG1	SYTL2
THBS2	BEX1	S100P	OBSCN	PRG4	STC1	CYP2S1	DUOX1	TMBIM4	TROAP	SPRY2	BRCA2
TMEM45A	MYH2	S100A2	STC2	MEOX1	GPR4	LDOC1	NR4A1	PABPC4L	TSPAN2	ITPR2	FKBP1A
ST6GALNA	MME	IGFBP4	PRKAR2B	TMEM178	CSF3	UACA	S1PR1	HES1	FGF18	STX3	HSPA2
IGHV3-30	IL1RL1	PYCR1	CAV3	CSRNP3	GPIHBP1	GNAI2	SLCO4A1	HSPB8	MTFR2	PTPN22	SGOL1
LUZP2	CDH13	P4HA3	SLC26A8	ABCC5	FOSL2	ITGA4	SLC26A2	SLC39A14	LOC73010	ADORA1	NQO1
NGFR	KLB	DDX3Y	IGF2BP1	TNNI2	PLA2G1B	BCL11A	MFAP5	SERPIND1	EPB41L2	CBLB	SMAD6
SCFV	FAM107A	MFAP2	STAT1	MDK	OSM	NR2F1	LCP2	ELTD1	LPAR6	CEBPB	NASP
NME9	GGTLC1	P2RY6	MYH6	RYR1	CD300LG	HNMT	SLC7A8	ARAP2	RNF182	UPP1	SMC2
FAM154B	ZNF385B	PLA2G7	SLCO4A1	IKZF2	CD274	KITLG	ADRA1A	RBP1	MATN2	CEBPG	PYGL
KRT13	LINC00165	DNM1	TRIB1	LGALS2	PDE4B	FABP4	CLASP1	DIEXF	PCDH18	ARHGEF3	NUP37
PLXNA4	LRRTM4	SNAP25	IL12B	MEOX2	S100P	HLA-DMA	MGST1	SQRDL	PLN	GHR	ECT2
IGHV3-48	HLA-DRB5	DSP	DAOA	ZNF518A	STK17B	ADRA2A	SPAG9	TNFAIP3	TRERF1	VLDLR	LIN9
SERPIND1	CADM1	FGF7	CD44	LINC01279	MAP3K8	RPS4Y1	SLC38A2	AHNAK2	BUB1	KRT19	CDK5RAP2

IPF stage 2 vs IPF stage 1											
GSE10667 - acute vs stable				GSE24206 - advanced vs early				GSE44723 - rapid vs slow			
Limma		NetWalker		Limma		NetWalker		Limma		NetWalker	
up	down	up	down	up	down	up	down	up	down	up	down
DEFA3	GSTT1	CA1	CXCR5	MT1M	RPS4Y1	IL6	ANGPT2	ITM2A	PTGFRN	CD3G	COL5A3
DEFA4	CD1C	CA12	GNAO1	APOLD1	DDX3Y	ZBTB16	ANGPT1	CDCA7	IL24	CD3D	COL5A1
OLFM4	FOSB	COL5A2	SCTR	S100A12	USP9Y	CEBPD	CXCL9	MYB	SRGN	ITK	MFAP5
SNORD14E	CD1A	COL3A1	ADCYAP1	SERPINE1	RTKN2	ATF3	PPBP	IKZF1	CD36	CD8A	FBLN1
SERPINB3	CCL17	HIST1H2BJ	GLP2R	RGS1	KDM5D	JUND	SSTR1	CMPK2	GPNMB	LCK	NOX4
SERPINB4	LOC100128	LOX	CRH	CXCL8	TXLNGY	FOSB	COL4A4	RAG1	MYO1D	PTPRC	TLR4
CRYAB	LHFPL3-AS	FGF7	MYH2	NR4A3	EIF1AY	FOSL2	COL6A6	LCK	HSD11B1	DEPDC1B	IL1B
HIST1H3D	FCER1A	DPP7	AMPD1	FKBP5	SOSTDC1	HP	ERAP1	TSPAN7	DPP4	ARHGDIB	IL1R1
ARG1	DDC	XRN1	SFTPA1	PER1	ZBED2	CD163	ERAP2	PTPRC	FAM167A	RHOH	ITGA11
S100A12	SERPIND1	S100A8	SFTPA2	SIK1	HLA-DRB4	FGA	CALCRL	TTK	CCDC102B	ARHGAP15	COL1A1
HIST1H3F	CCR7	S100A9	DMBT1	ZBTB16	ZFY	ARG1	GNAS	ARHGDIB	TEK	TTF2	FOS
HIST1H2AM	WIF1	SOD2	RASGRF1	CSRNP1	NR5A2	MXD1	TEK	HMGB2	DIO2	MYB	DLL1
HBD	LGALS2	MPO	GRIN2A	GSTT1	HOXC6	MYC	P2RY13	MND1	IL11	KIF4A	NOV
LINC01127	KLHDC8A	SLC4A1	BAAT	ADAMTS1	SLC40A1	SOCS2	KBTBD7	CRNDE	SERPINB2	CCNA2	PTGDS
PROK2	CD1E	CFTR	AGTR2	FAM216B	FFAR4	IRS2	ZNF24	TOX	POSTN	CDK1	PTGS1
SLC7A11	CPA6	SLCO4A1	CXCL11	S100P	CTSZ	NNMT	CCR5	NEIL3	CYGB	CDT1	FBN2
NPPA	CRH	ALAS2	CCR7	NR4A2	MS4A1	NAMPT	COL3A1	BUB1	FAM155A	HERC5	FBN1
PGLYRP1	IGF1	KYNU	CCR6	C20orf85	UTY	ARG2	HLA-DRB5	BCL11B	GRIK2	PBK	CXCL5
LOC344887	KCNH2	CCL20	CYP2C18	MT1X	LOC642236	CXCL6	HLA-DQB1	HPGD	EPHA5	SH2D1A	IL8
HSPA1A	CIDEA	ARHGEF12	GSTT1	MT2A	PPP1R3C	IL8	TRBC1	LMNB1	AREG	PRKCB	SSTR1
RSRP1	SFRP5	CASR	PLA2G3	ERICH3	ANKRD12	CXCR4	IL1A	ADA	CLEC2B	DTL	CDKN2A
BPI	SHISA2	DEPDC1	ENTPD3	C9orf135	NREP	BDKRB2	PRKG1	AIM1	AADAC	WDR76	RIN2
HILPDA	SKIDA1	CEP152	TPO	CAPSL	CBLN4	CCNA1	PPP1R12B	ARHGAP15	PTPRE	CCNB1	CYP1B1
ALDH1A3	CHI3L1	NEK2	DDC	SLC19A2	CAMK2N1	FOSL1	CASP1	DEPDC1B	STEAP2	TOP2A	HSD17B2
KCNQ1OT1	GRIN2A	CENPI	FOS	DYNLRB2	TIMD4	THBS1	RBM26	CD3D	THBS2	LMNB1	COL1A2
HIST1H3B	C1orf186	CENPE	TGFB2	FPR1	FDCSP	NFIL3	CCR2	SEPT6	BOC	PNMA2	SEMA3A
MS4A6E	LRRC55	KIF2B	HDC	CLEC4E	LGALS2	CREM	TLR8	C2orf40	SERPINB9	CYFIP2	DPYSL4
PHLDB2	ODF3L1	PDE4D	CHIT1	SLCO4A1	COL6A5	COL6A1	TLR7	CORO1A	ALDH1A3	HMGB2	TEK
MYO7A	CALB2	GUCY1A2	CHIA	CAPS	GIMAP7	CXCL1	CXCL11	RHOH	PTGS1	RAG1	PTPRB
PDGFRA	BAAT	SART3	UGT2B7	CDHR3	NR2F2-AS1	SLC27A2	SP3	SLAIN1	CCL2	UBASH3A	CD36
SLCO4A1	KRT1	MEGF10	FOXA2	DUOX1	SEMA6A	ACSL1	TNFSF10	KIF15	ADAMTS12	PRIM1	HSD11B1
ZFX	XCL1	CASC5	HLA-DQA2	MFAP5	OVOS2	ARHGDIA	IRF8	CDK1	EDNRB	MCM10	SNCA
GUCY1A2	NELL1	ABCA1	KRT1	IL1R2	TM4SF18	RHOB	CYBB	PBK	NOV	CDC7	GABARAPL
HMOX1	RGS13	DLC1	CALB2	CEBPD	ID2B	IL6ST	NFYA	SPC25	HSD17B2	POLE2	A2M
ZNF462	CDH16	CDC5L	PENK	IL6	HOXA3	SLC11A1	LCK	PVRIG	LOXL2	CDC6	THBS2
RNASE3	PGC	DISC1	SRP9	NAMPT	HOXB3	CXCR2	MS4A1	TOP2A	PTGDS	KIF11	COL3A1
PFKFB3	LRRN4	NPPA	RPL9	MT1H	CCR2	SELE	PTPRB	KIF20A	TOR4A	BUB1	SDC2
SEC24A	ZNF319	HNF4A	CITED1	CCDC78	DNASE1L3	NR4A1	EDNRA	HERC5	VAT1L	CEP55	CEBPB
MS4A14	DUSP27	MGAM	TFAP2C	NR4A1	CCL8	SERPINE1	SLC9A1	NDC80	KCND2	CDKN3	DSP
MMP8	HDC	SLC5A3	SFTPD	MT1G	HIVEP3	SLC1A3	PTPN22	CDT1	TENM3	CCNB2	CSTA
DISC1	C16orf89	FNIP1	HLA-DQA1	AREG	TCF21	SLC7A11	IL2RB	THEMIS	SOCS3	HCLS1	EGR1

GNB5	S100B	FLCN	EIF5	C9orf24	LINC01094	PIK3R3	SYK	HCLS1	DPYSL4	TAL1	AXL
UBE2B	C2orf54	ZP2	SERPINA1	LRRC46	LRRN3	RGS1	SPNS1	FAM83D	TNFRSF11E	GATA3	SHC3
ADCY10P1	APOH	ZPBP	CTSG	MT1HL1	COL4A4	PXN	CCR1	NUF2	SPON2	VAV3	CASP1
SAA2	PA2G4	CCL16	SERPIND1	MT1E	QKI	PDE4B	PRKY	KIF11	TMEM98	CCNE2	BDKRB2
PGAP1	CHIA	SERPINB13	CACNA2D2	C11orf88	GUCY1A2	AK7	GRIA1	ITK	LAMA1	PRC1	LAMA1
SYCP2	HSPB3	CTSB	VPRBP	PDK4	NTM	GFPT2	HLA-DQA1	CCNB1	SGIP1	CMPK2	LAMB1
NPHS2	TPO	RUNX2	NR4A1	C1orf194	TLR7	CXCL2	BCL11B	KIAA0101	ELMOD1	RRM2	PRDM1
F2RL3	SELE	TLE1	NTRK1	AZGP1	CR2	MYH11	NR2F2	HMMR	CDKN2A	CENPF	ATXN1
THNSL2	IGFL2	TERT	CAV2	ROPN1L	GPR18	KLF4	SH2D1A	FANCD2	PDGFD	AURKB	IL6
ESM1	CXCL13	PRKG2	MALL	ATF3	BMP5	KLF6	ARHGAP29	CENPA	FAM105A	NDC80	PTGS2
HBM	VGLL1	RUNX1	TNNC1	TMEM190	TMEM255A	NFKB2	USP9Y	KLHL23	COL5A3	MLF1IP	LAMA2
MGAM	LPPR1	PAPPA	TNNT2	WDR38	HOXA5	NFKBIA	ST8SIA1	UBE2C	SHC4	NUF2	DPP4
LSMEM1	CCR6	SKIL	IGF1	MT1F	LINC01268	REL	ST3GAL6	GATA3	TMTC1	CENPH	FAP
VIP	CAPN6	PIP	IGFBP5	TEKT1	HLA-DQB1	MMP9	BCL11A	PRR11	IGDCC4	SPC25	FGF7
CA1	LOC10106(	EEA1	CDH3	ARG2	SP3	SLC7A2	PRKACB	RAG2	COL3A1	CENPA	CTSB
RUNX1	XAGE2	NUF2	CDH1	MGAM	PRKY	ALOX15	HTR2B	TMSB15A	BMP2	DEPDC1	PLAU
HIST2H2AA	TMED6	CCNA2	HLA-DRB5	BRE-AS1	LOC643733	PLA2G2A	GNG2	CDKN3	PTPRB	LEF1	SERPINB2
NEK2	C4BPA	CDC25B	DRD5	DDIT4	FLJ34503	MGAM	GZMA	CCNB2	ZNF295-AS	ADA	F3
CRISPLD2	HLA-DQA2	CGA	NR0B2	FPR2	GIMAP6	SLC2A14	LMNB1	PLCL1	CNIH3	TMPO	TFPI
KIF11	SUSD4	NSF	MYC	ADAMTS9	TRPC6	TGM2	NFYC	GINS1	SNAI2	RAG2	MEGF6
LEP	CD207	RGS9	PLCB2	C4orf22	HERC2P3	HSPD1	ALOX5	CD8A	CDCP1	CD1D	BMPER
FGF7	PCSK2	GNB5	AMBP	C1orf192	DDIAS	ACSL4	TBL1XR1	NUSAP1	RARRES2	NCAPG	BMP2
STC2	NAPSA	GRM7	MS4A2	HSPD1	NBR1	PTPN11	FCRL3	TPX2	PRDM1	UBE2C	KITLG
SMC5	DMRTC1	TP63	FCER1A	EFHB	RGS13	ALOX15B	TOP2A	DLEU2	SHC3	FANCD2	SNAI2
CCNA2	MEOX1	DERL1	CXCR3	SPAG6	GZMA	PIP	RPS4Y1	NLRC3	TLR4	BLM	SERPINB9
CCDC57	SERTAD4	HSPA12A	FOXA1	KLF9	HHEX	SPI1	EEF1D	PRKCB	ARHGAP6	SKP2	CASP4
HDAC10	YTHDC1	FGFR2	ALDH1A2	FAM81B	TRAF3IP3	CREB3L1	GABPA	FAM169A	CPE	CDCA8	RUNX2
BCL2L11	DCANP1	ASPM	SLC22A3	MMP19	LAMA4	IL6R	MS4A2	ZIC2	ELFN2	HMMR	SPP1
SLC16A10	AADAC	PDGFRA	IGFALS	C6	CDKN1C	SOCS3	FCER1A	CD1E	DOK6	TPX2	EGFR
PDE4D	CAPRIN1	PTK2	BDNF	CFAP53	CTNNAL1	AREG	LAMA4	AURKB	FAM162B	IKZF1	SDC4
AHSP	CAPN8	CENPA	MYCN	ERRFI1	LINC00622	B4GALT1	PTN	ARPP21	KLF9	IKZF2	IGF1R
SPATA8	IFT57	CCNE2	SLC1A2	PADI4	BANK1	SAP30	SLIT2	MCM10	NOX4	BIRC5	SOCS3
SOST	CD1B	GPX3	ADRB2	PIFO	GNAS	FOXO1	SLAMF7	JPH1	GDF15	FANCI	SHC4
HUS1B	SF3A1	PAH	SHC3	IRS2	GIMAP1	CCL20	ARL15	HHIP-AS1	IL13RA2	DNA2	NID1
NPL	HABP2	SLC16A10	MIP	AK7	HTR2B	S100A8	GUCY1A2	DPY19L2P2	ANKRD28	NCAPH	PDGFRB
HIST1H2AE	CPA3	NDC80	TPM1	SPIDR	LOC100289	S100A9	GUCY1B3	PAX6	PSG5	RBL1	JUN
GPER1	SLC6A20	VIP	ATP1A2	MXD1	SIGLEC6	FN1	ANKRD12	KIF4A	LOC100132	KLHL13	CXCL3
FATE1	ECEL1	ADM	SLC18A2	NR1D1	ND6	SCGB1A1	PIK3CG	CEP55	F3	MAD2L1	MGP
MPO	ENY2	F2RL3	LDLR	SYTL3	FRAS1	SLC16A10	GATM	CCNA2	CYB561	TACC3	COL7A1
LPIN1	NAPSB	F2RL2	PCSK9	PTX3	DTL	SLC7A5	SLC15A2	ZNF423	PNMAL1	CXCR4	FBLN2
TMEM100	PCSK9	CDC14A	NKX2-1	ARG1	ANKRD20A	IL1B	JUN	CENPU	ICAM1	EPB41	IL13RA2
ALK	MS4A2	KIF20A	DPP4	SLC44A4	PLK2	IL1R2	ADCY7	PSMB9	SSTR1	ARHGAP19	ICAM1
XRN1	SIAE	ARG1	CXCL2	RHOB	CLEC12A	PPP1R15A	DST	OIP5	MANBA	FOXM1	CSF2
ASPM	ENDOU	PRKCE	MET	ZFP36	LINC00936	RHOBTB2	BLNK	TRAF3IP3	LAMA2	MKI67	COL5A2
VNN1	ZBED2	CRYAB	CCL11	C10orf10	RUNX1T1	STAT3	SLC18A2	TYMS	FOXQ1	BARD1	PDGFRA
DNASE1L2	TRIM66	HIST1H2AD	SLC15A2	C11orf70	EMCN	IBTK	HNMT	ATP8A1	CABLES1	PITX2	PLA2G4A
HIST1H2AD	EMP1	ABL2	ATP1A4	LOC100505	PTGDR	RGS2	PTPRM	CYFIP2	KCNJ15	TYMS	BDNF
S100A8	IL22RA2	IL3	POLR3H	DNAJA4	PPFIBP1	ELF3	PPFIBP1	ASPM	RCN3	DUT	CPE
WDFY4	RORC	ACSL1	ICAM1	MAFF	THAP2	NFATC1	HOXA5	ITGB2	INHBA	MCM6	IL1RAP
ZNF765	LOC100128	PGAP1	SLC6A20	APOBEC3A	CAT	LMNA	MEIS1	LDLRAD4	STEAP1	AURKA	FGD4
B4GALNT1	THRSP	MYBL2	TNN	ALPL	CYP39A1	GLS	KBTBD6	P2RY8	GNG11	CASC5	ARHGAP6
RRN3P2	VWC2	ARHGAP11	SDC4	MYC	LPPR4	IL20RA	PTGDR	PRIM1	F2RL1	DEF6	CXCL1
S100A9	RIMKLA	HSPA6	KIT	HP	FCRL3	FGG	NOSTRIN	SCN2A	ERO1LB	TFDP2	CARD16
RNF213	KCNF1	DEPDC1B	GRAP2	ZMYND10	LINC00667	CSF3R	CAT	SH3TC1	LOC100128	GINS1	SEPP1
CENPE	GLP2R	VIPR1	NFATC2	ARMC4	TRD	CEBPB	HHEX	MBNL3	ANTXR2	ZAP70	IRAK3
SLC10A7	VWDE	RABGAP1	ABP1	CXCR4	LOC389834	EPHX1	FGFR1OP2	CDKN2C	SLC22A15	CDC25A	GREM1
TBX6	GPC5	DNA2	COL17A1	TSC22D3	ZKSCAN7	GSTT1	TRAF3IP3	POLE2	CEMIP	DHFR	PVRL3
EEA1	SFTPA1	CPS1	LAD1	FAM107A	ZNF780A	NR1D1	EFNB2	MFNG	LACC1	ARHGAP11	CLDN1
NFE4	DRD5	ZFYVE16	CD3D	RGS2	CX3CR1	NR1D2	RASGRP3	CHD7	SEMA3A	KIF15	LAMA4
DUSP5P1	SVOPL	SERPINC1	CCL17	RSPH9	NLRC3	SHC1	C4BPA	CENPH	TGIF1	CENPE	CCL20
RUNX2	CRTAC1	PDLIM5	VCAM1	KCNE1	RNF182	SPAG9	BMPR2	WDR76	WISP1	NASP	MPDZ
MAGEB1	UGT2B7	ALK	LRP8	CCDC181	LGR5	SLCO4A1	KLRB1	BACH2	RIN2	MAP2K6	TRIO
FAM95B1	SHISA6	ADORA3	APOH	DNAI1	FCER1A	SLC4A4	CLEC2D	KLHL13	DSP	FBXO5	CES1
CASR	CRYM	DLEU1	S100B	DUSP1	ARL15	SGPP2	PDE7A	NCAPG	RGCC	MCM8	RASGRF2

CCDC93	PPIL2	KNTC1	GFAP	FGA	LOC283788	PPAP2C	PTPRG	LEF1	CDH11	CHEK1	IL1A
CEP152	ENTPD3	MXD1	IYD	LRRC34	TYRP1	FASN	HDAC9	AIF1	ARNT2	MCM2	IGFBP5
CCDC88B	TNNC1	RAB24	PARD6B	C9orf116	DNM3OS	FAM107A	NR2F1	SH2D1A	CORIN	SMC4	NR2F1
ZNF695	SCTR	LHCGR	TP53	KLF4	DMRT2	KRT15	SOS1	UBASH3A	DIRC3	EXO1	ITGA1
RNASE2	MT1E	MED14	KRT18	CREM	THEMIS	RHPN2	PTPRC	PPM1E	LINC00674	RFC3	ROBO2
TMOD2	TRERF1	NECAB2	KRT8	WDR78	ANKRD29	C10orf10	CIITA	HHIP	LAMB1	RACGAP1	GNAI1
PAPPA	SORCS2	STAM2	PON3	PIH1D2	B3GALT2	COL1A1	GSK3B	TTF2	RORA	PTPN2	SPHK1
ESCO2	KRT8	SLC2A3	C5	RASD1	TRMT13	RELA	CCNA2	CDC7	GABARAPL	STMN1	SRPX2
COL5A2	CCDC87	QKI	TRIM63	SYBU	CCDC88A	TSC22D3	GUCY1A3	NRN1	KLHL5	LBR	PTPRJ
PLOD2	PROC	C10orf10	HSD17B8	IGFBP2	BCL11B	JUNB	EHHADH	DTL	IL1R1	ZWINT	ROBO1
SERPINC1	DIRAS3	HSP90AA1	CTGF	CHI3L1	GIMAP2	SLC2A3	CACNA1D	STK26	PEAR1	SHCBP1	PLAT
PKP1	CYP2C18	BUB1	PTPN3	PELI1	FBXL3	PLA2G10	WNT5A	RNASEH2B	TMEM52B	POLA1	PDGFD
CLCA4	SFRP1	SLCO1B1	BBOX1	CCDC173	GZMH	RHOU	FZD8	BIN2	DHRS3	PTPRCAP	JAK1
ATP13A3	KRT27	NKX2-2	HSPB3	C9orf117	LTB	S100P	PREX2	CD3G	COL1A1	SPC24	CGA
LOC157562	RAB33B	OLIG2	TPM3	BDKRB2	LPAR6	EZR	TGFBR1	PRKCQ-AS1	SCG5	SPAG5	HPRT1
ZFPM2	DACT2	RXRA	CXCL13	PROK2	RAB30	TFE3	GRK5	EPHB6	WIPI1	CENPM	PDE4B
FLCN	TGFB2	CREM	PACSIN1	FOSL2	FREM3	MAFK	APOBEC3G	CD53	SRPX2	BRCA1	MAP3K5
ZNF876P	BTC	MYL1	RTKN2	MUC5AC	PPP1R12B	TNFAIP6	IFIT2	KIR3DL2	CCDC85A	RFC4	AK5
SLC4A1	SFTPD	MYBPC1	CUL3	MAP3K19	NAPSB	FOXO3	IFIT3	HELLS	FLJ35700	MELK	PAPPA
LOX	MAGOHB	RAD23B	PTGS1	NFKBIA	HAS3	GSTA3	RASGRP1	PLK4	FGF7	PLK4	GREM2
KIF15	C2CD4B	NGLY1	MMP7	CLDN3	ITGB1BP1	FPR2	GRB14	FLJ38379	TMEM45A	NEK2	TNFRSF11B
PCID2	TSPEAR	KCNE4	HLA-DQB1	CYP2B7P	ARHGEF28	NAB2	RAMP3	CENPF	CTSB	NCAPG2	LYN
TMEM87B	APOBEC2	KCNA1	CYP4B1	SPSB1	GAPT	C5AR1	VIP	DOCK2	SHROOM2	RFC5	LAMC1
CDH24	FHDC1	TYR	SLC7A10	DLEC1	CCL23	SERPINB2	CASP8	TAL1	LDLRAD3	SKAP1	PTGES
SPESP1	FOXA2	ONECUT1	FOLR1	IL18RAP	ZNF404	PLAUR	XPO1	NCAPH	WNT5A	MEF2C	LY96
ZFY	TDGF1	LIN7A	FOLR3	LOC100288	CYS1	CTNNB1	YWHAH	RRM2	MXRA7	RAD51	AREG
TTK	HHIP	FOXM1	PLA2G1B	VNN3	PHOSPHO2	GCLM	CCDC88A	TMPO	SLC16A6	USP1	COL6A2
DLC1	CCDC178	CXCL6	ESR2	C11orf97	RBM15	PIAS3	ALOX5AP	CENPW	NTM	PRKCQ	CTBP2
NEAT1	MYH2	MELK	RSPO1	DUSP5	SEMA6D	LCN2	DEPDC7	CDC6	CD248	SATB1	TGIF1
GNRH1	DNASE1L3	MYH8	DKK1	SLC7A11	LOC100506	IL1R1	TIAM1	KIAA0922	DAB2	MCM3	KRT7
HIST1H3H	GREM2	UBE2B	CCL19	CYP4F3	PCDH18	GADD45B	PPP1R3C	HJURP	IGFBP5	ELMO1	CACNA2D3
CEMIP	ADIPOQ	CDKN1B	ACOX1	ITPKC	CYBB	CDKN1A	PHKB	NPNT	FOS	DOCK2	RASGRP3
ARHGEF12	PACRGL	IL12B	SCD5	CNIH4	SESN3	RARA	SMAD1	PRC1	C2CD2	MAP4K1	MITF
SNX5	C3orf70	IL12A	FXYD3	NNMT	ANKRD20A	NR4A2	PDE5A	KIF18B	PXDN	KIF2C	SQSTM1
PBK	FMO9P	CALCRL	CLN5	ZNF331	POSTN	ABL2	ARRB1	FOXM1	VSTM4	MCM5	TGFB2
C2orf88	CHRNA1	GOSR1	GLI1	GM2A	DHX29	JDP2	FASLG	CDCA3	UGGT2	UBE2T	IRS2
RFPL3S	DMBT1	PKP1	HHIP	VNN1	SAMD3	XRCC6	EGR2	CCNE2	TPST1	MCM7	UPP1
CENPI	ADAMTS8	CCDC88A	SDC1	SPEF2	BCL11A	PDE4D	CD48	PDE3B	TPBG	KIF23	ALDH1A1
LYG1	C1orf132	FGB	THBS4	JUND	ITPKB	MMP1	CD2	BCL11A	STC1	SGOL1	CEBPD
YOD1	UBD	SCGB1D2	KRT27	TNFAIP3	PTPRB	SOX17	CDKN1C	KCNQ5	LDB2	HMHA1	CXCL2
DEPDC1	LIFR-AS1	SCGB2A2	KRT7	VNN2	MS4A2	NEDD9	BICD1	VAV3	DGKI	CENPK	ITGA2
RAB42	XCL2	TRIP13	INADL	CX3CL1	TNFSF10	CCND3	SOX4	SKP2	FAM198B	POLQ	IL6ST
PPIG	GRAP2	SEC24A	SFRP1	CXCL1	LOC100134	SPAG16	MYCN	CNKSR2	ARHGEF28	ALDH5A1	PTGIS
HIST1H2BJ	HIGD1A	RHOT1	WIF1	THBS1	KBTBD7	SPAG6	HIST1H2AC	BIRC5	TFPI	OXCT1	OAT
MKNK2	EXPH5	CREBBP	WNT7B	CD163	MIR155HG	DGAT2	APLNR	ST8SIA4	EMP1	PTTG1	KYNU

Supplementary Table 2: All lists containing the important up- and down-regulated genes for each stage. Genes that were found in all three datasets per analysis are highlighted in light gray.

IPF vs normal		stage 2 vs normal		stage 1 vs normal		stage 2 vs stage 1	
up	down	up	down	up	down	up	down
COMP	EDNRB	SERPINB3	FOS	COMP	CEBPD	ARG1	FCER1A
SPP1	CEBPD	KRT6A	IL6ST	SPP1	CYP3A5	S100A12	LGALS2
SCG5	CA4	MSMB	EDNRB	RPS4Y1	PLA2G1B	PROK2	RGS13
COL1A1	PLA2G1B	COMP	RTKN2	SCG5	ADRB1	SLC7A11	C4BPA
MMP1	AGER	MMP1	FOSB	IGF1	MT1E	SLCO4A1	NAPSB
KRT6A	GRIA1	BPIFB1	HSD17B6	FNDC1	STC1	MGAM	MS4A2
BPIFB1	BTNL9	SPP1	HHIP	SERPIND1	IL6	SLC16A10	ZBED2
KRT5	HHIP	COL17A1	TNNC1	MMP1	APOBEC3A	PDE4D	DNASE1L3
MMP10	CLDN18	KRT5	CLDN18	KRT6A	AFF3	VNN1	CXCL11
UGT1A6	CCK	CXCL6	PLA2G1B	UGT1A6	GPIHBP1	S100A8	HLA-DQA1
GPR87	CYP3A5	COL3A1	AGER	KRT5	IL1RL1	S100A9	HLA-DRB5
IL13RA2	ADRB1	LCN2	CA4	IL13RA2	FAM107A	CCL20	MYCN
PLEKHS1	EPB41L5	PLEKHS1	CPB2	GPR87	ZNF385B	PIP	SLC18A2
KRT14	FIGF	CTHRC1	EPB41L5	MMP7	IL6ST	ABL2	SLC15A2
GREM1	PEBP4	PROM1	PEBP4	KRT14	IL6R	ACSL1	RTKN2
KRT17	IL6	S100A2	CCK	KRT17	CXCL3	MXD1	HLA-DQB1
MSMB	FOSB	MMP10	ADRB2	SFRP2	CXCL2	SLC2A3	AADAC
MMP7	GPIHBP1	KRT17	ZNF385B	COL1A1	ADAMTS1	C10orf10	EMP1
CXCL6	ADRB2	SIX4	GPIHBP1	HS6ST2	SLC6A14	CREM	TGFB2
LCN2	ZNF385B	GPR87	EMP1	CDH3	CD274	CXCL6	GREM2
PROM1	CHRM3	KRT14	GRIA1	CXCL14	NFKB2	NEK2	FOS
S100A2	STC1	COL10A1	SDR16C5	KRT15	PTGS2	KIF11	IGFBP5
SIX4	CCDC85A	CP	CHRM3	CXCL13	ZBTB16	CCNA2	BDNF
CTHRC1	ACADL	UGT1A6	ACADL	S100A2	HMGCS1	ASPM	SHC3
COL10A1	IL6ST	THBS2	SLC39A8	COL10A1	STAT3	CENPE	DPP4
SFRP2	IL6R	CLCA2	PGC	CTHRC1	S1PR1	KIF15	CXCL2
POSTN	CXCL3	FAM216B	IL6	DIO2	CSF3R	TTK	ICAM1
HS6ST2	SLC6A14	COL1A1	LDLR	MMP16	CEBPB	PBK	SDC4

CP	PTGS2	ZBBX	CYP3A5	COL3A1	SOCS2	DEPDC1	PTGS1
COL3A1	ADAMTS1	SCG5	CXCL3	GSTA1	LIFR	CASC5	KRT7
CLCA2	SOCS2	CFAP53	DLC1	PCDH7	PFKFB3	NUF2	NTM
KRT15	LIFR	DYDC2	PTGS2	TDO2	SLC2A3	CENPA	LAMA4
IGF1	CEBPB	C20orf85	ADRB1	THBS2	KLF6	CCNE2	ARHGEF28
GSTA1	LIF	GREM1	IL6R	ST6GALNAC1	FLT1	NDC80	POSTN
DIO2	SLIT2	TTC29	SLC1A1	CYP2F1	SLC2A14	KIF20A	PTPRB
CDH3	ZBTB16	MUC16	TIMP3	TP63	IL8	ARHGAP11A	SSTR1
CXCL14	S1PR1	SPAG17	MYC	EPHA3	STC2	DEPDC1B	TEK
SOX2	CD274	ARMC3	SLC6A14	CCL13	SLCO4A1	DNA2	COL3A1
CXCL13	FAM107A	IL13RA2	NCKAP5	CXCL5	TMSB15A	BUB1	IL1A
THBS2	STAT3	GSTA1	CAV1	COL7A1	MATN3	FOXM1	CASP1
CHST9	KLF6	TDO2	CHRM2	TF	PTN	MELK	JUN
PCDH7	PTPRB	C6	TGFB2	COL1A2	PRKAR2B	CXCR4	RASGRP3
TDO2	SLC2A3	C11orf88	SOCS3	IGFBP2	MAOA		NR2F1
FAM216B	CSF3R	MMP7	ST6GALNAC3	CCL19	AOX1		WNT5A
SIX1	MATN3	CXCL12	EGR1	PLN			
DYDC2	MME	WDR16	ARHGAP6	CXCL12			
MUC16	CHRM2	C9orf117	GALNT15	COL5A2			
CYP2F1	SHANK2	MUC4	MAOA	IGFBP4			
ALDH3A1	PTN	CCDC170	RNF182	DDX3Y			
CCL13	CREB5	ABCA13	AOX1	MFAP2			
TP63	TEK	SPAG6	TFPI	DNM1			
COL7A1	SOCS3	MS4A8	BTG1	GREM1			
CXCL5	NR4A2	C1orf194	GPX3	CDH2			
IGFBP2	ST6GALNAC3	MAP1A	TNFRSF11B	HTR2A			
MMP2	MAOA	KRT15	CDKN1C	IGFBP5			
COL5A2	GPX3	IGF1	NR4A2	FBLN2			
COL1A2	AOX1	DST	FGF2	ITGA11			
ALDH1A3	PDE4B	SIX1	NID1	LAMA1			
FABP6	TFPI	MMP2	TEK	MFAP5			
ALOX15	CD44	CYP2F1	EPAS1	GSTT1			

TF		COL5A1	CD44	DSP			
COL5A1		IGFBP2	PDE4B	NTM			
MFAP2		COL1A2		MGP			
ABCC5		ALOX15		PTGFRN			
PDE1A		MFAP2		IL33			
AGBL2		SLC16A10		SULF1			
KCNMA1		PSAT1		EIF1AY			
CCL21		MFAP5		COL5A1			
CCL19		DEPDC1B		CPE			
DNM1		CDT1		IGFBP7			
ABCC3		DDX3Y		NTS			
RPS4Y1		KLHL13		GPX7			
HTR2A		CPE		PLA2G4A			
PSAT1		MYB					
CDH2		FN1					
NGEF							
MFAP5							
IGFBP5							
SLC16A10							
FNDC1							
MGP							
SULF1							
CPE							
IGFBP7							
MYB							
SFRP4							
KRT19							

Supplementary Figure 1: Structural distances from FDA approved drugs-IPF vs Normal

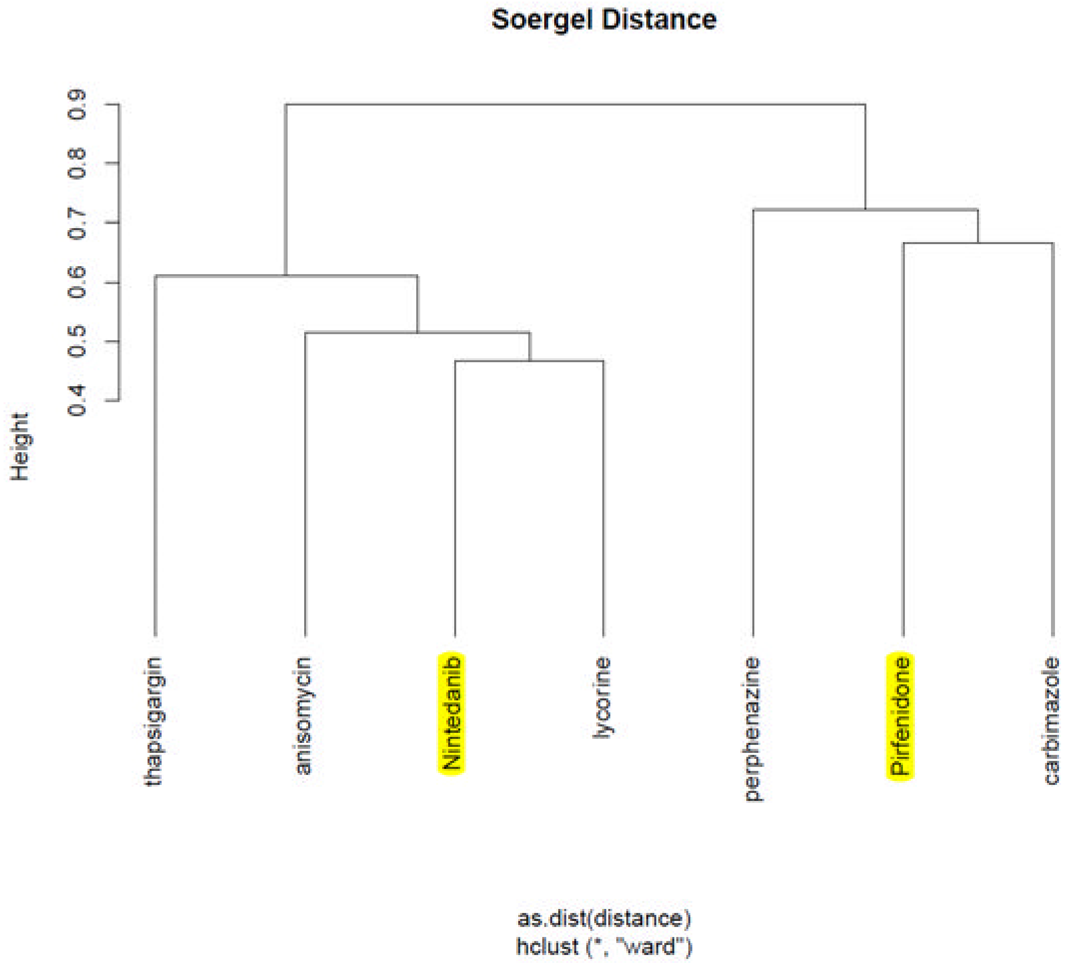

Supplementary Figure 2: Structural distances from FDA approved drugs-stage 2 vs Normal

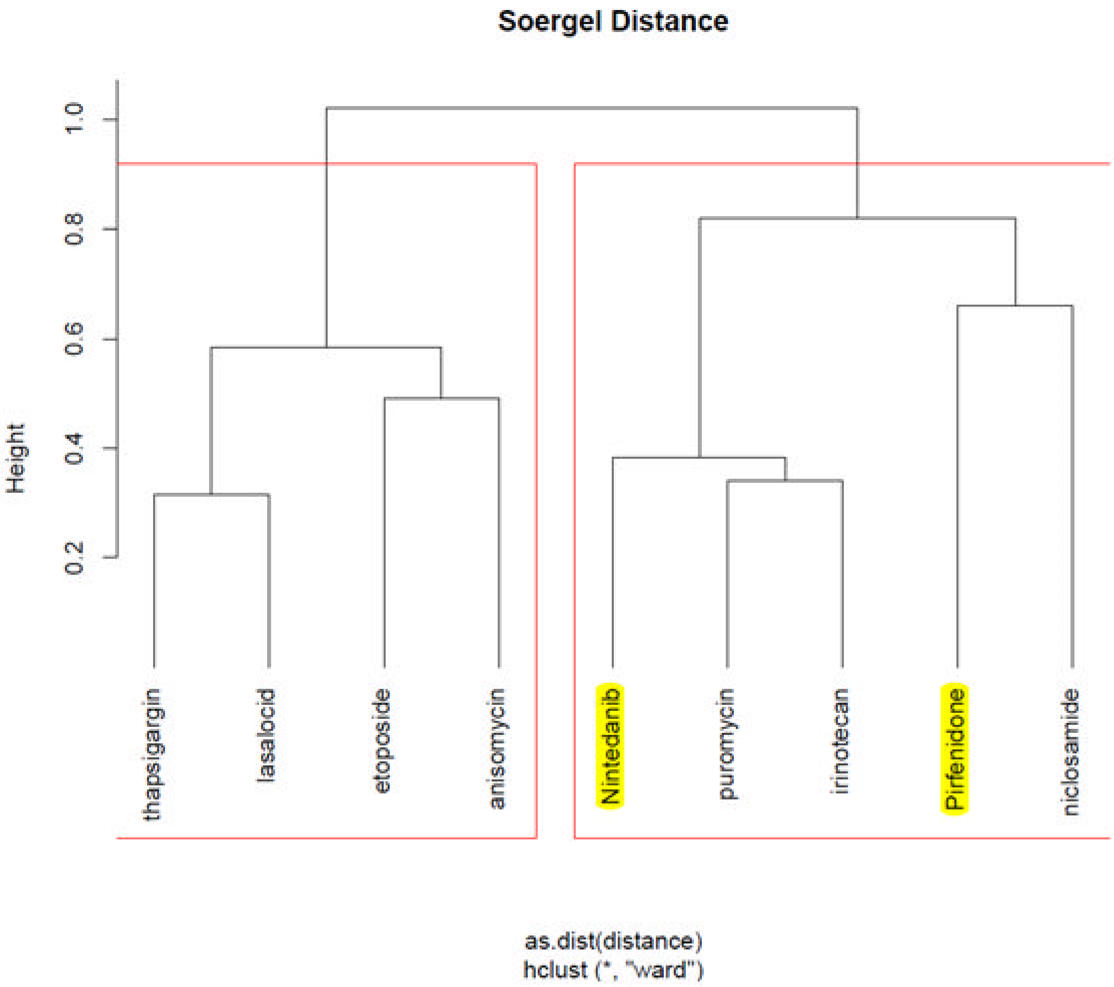

Supplementary Figure 3: Structural distances from FDA approved drugs-stage 1 vs Normal

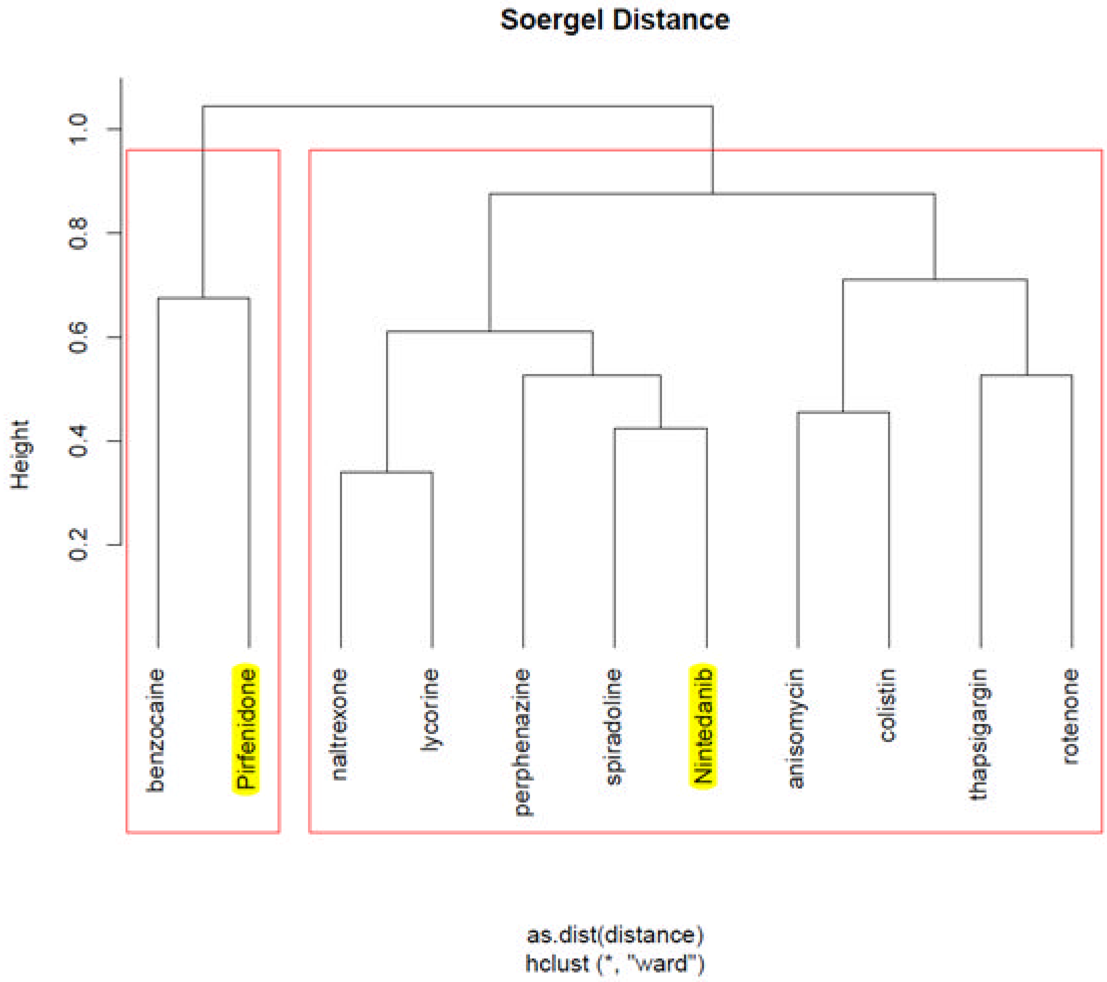

Supplementary Figure 4: Structural distances from FDA approved drugs-stage 2 vs stage 1

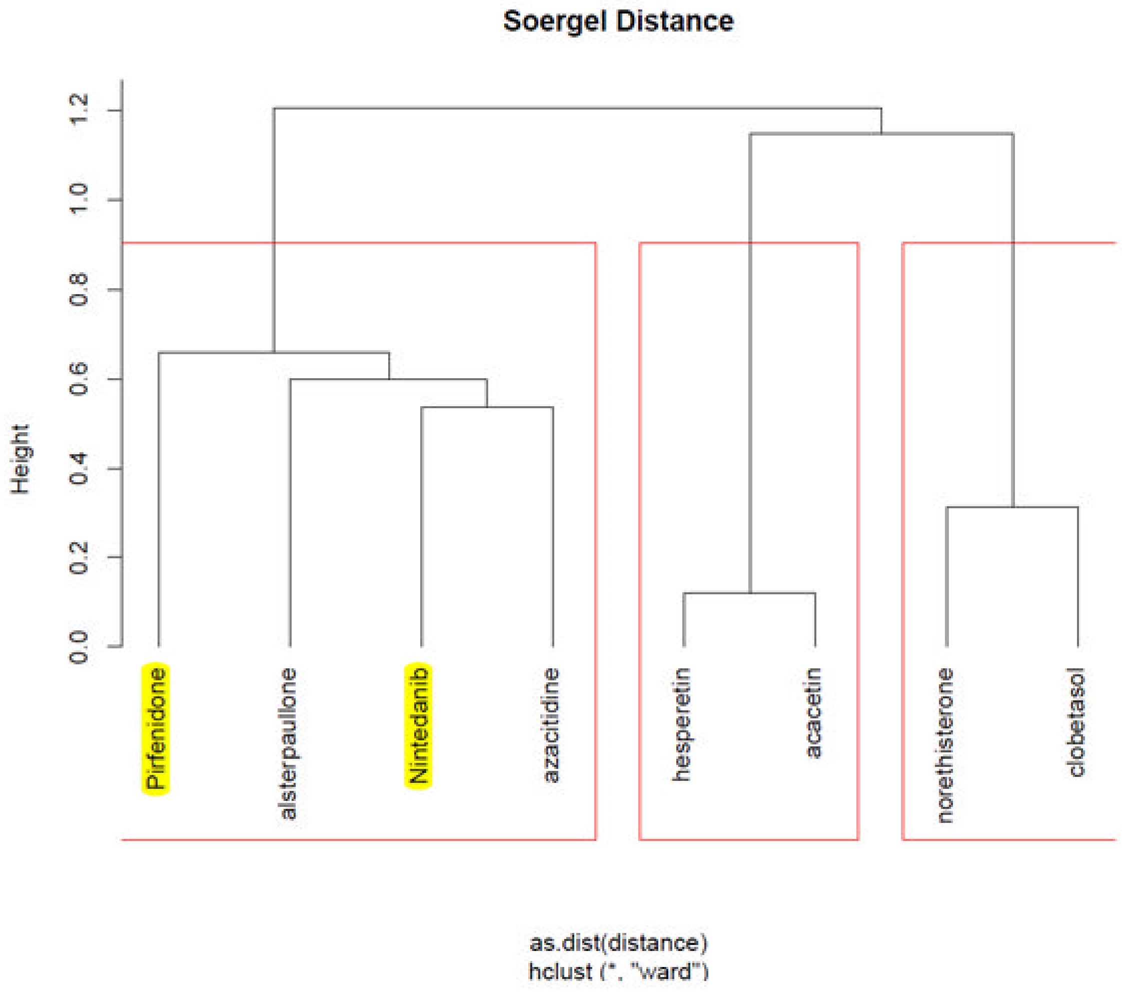

Supplement: Supplementary file 1 — Supplementary Information [file 41598_2017_12849_MOESM1_ESM.pdf]
